# Long‐term safety of dietary salt: A 5‐year ProspEctive rAndomized bliNded and controlled stUdy in healThy aged cats (PEANUT study)

**DOI:** 10.1111/jvim.16952

**Published:** 2023-12-12

**Authors:** Brice S. Reynolds, Valerie Chetboul, Jonathan Elliott, Jeremy Laxalde, Patrick Nguyen, Isabelle Testault, Laëticia Dorso, Jérôme Abadie, Hervé P. Lefebvre, Vincent Biourge

**Affiliations:** ^1^ InTheRes, Universitéde Toulouse, INRAE, ENVT Toulouse France; ^2^ Unité de cardiologie d'Alfort (UCA), CHUVA Ecole Nationale Vétérinare d'Alfort Maisons‐Alfort France; ^3^ Université Paris‐Est Créteil, INSERM, IMRB Créteil France; ^4^ Department of Comparative Biomedical Sciences, Royal Veterinary College University of London London UK; ^5^ Royal Canin Research Center Aimargues France; ^6^ Nutrition and Endocrinology Unit, LUNAM Université Oniris Nantes France; ^7^ Atlantia Veterinary Hospital Nantes France; ^8^ Centre hospitalier Univesitaire Vétérinaire Oniris Nantes France; ^9^ LabOniris, Oniris Nantes France; ^10^ Université de Toulouse, ENVT Toulouse France

**Keywords:** blood pressure, cardiovascular health, glomerular filtration rate, kidney health, sodium chloride, urolithiasis

## Abstract

**Background:**

High‐salt diets promote urine dilution and decrease urolithiasis risk.

**Objective:**

Prospectively evaluate the safety of chronic high dietary salt intake (randomized controlled trial).

**Animals:**

Twenty research colony neutered, healthy aged cats (11.5 years [10.0‐11.6], median [interquartile range]).

**Methods:**

Healthy cats were randomized to control or high‐salt dry diets (sodium: 1.02 ± 0.16 [mean, SD] and 3.26 ± 0.30 g/Mcal metabolizable energy [ME], respectively; chloride: 2.26 ± 0.33 and 5.71 ± 0.28 g/Mcal ME, respectively), fed for up to 60 months. Assessments included CBC, plasma biochemistry, urinalysis, glomerular filtration rate (GFR), blood pressure, renal and cardiac (conventional Doppler and 2‐dimensional color tissue Doppler) imaging, annually. Cats that died or were euthanized underwent necropsy. Diet effects over time were evaluated with linear mixed models.

**Results:**

Follow‐up duration (median [Interquartile range]) was similar between the control (38.7 months [28.6‐48.2]) and high‐salt group (51.4 months [45.7‐59.0]). Diet had no significant effect on changes in GFR, blood pressure, plasma creatinine concentration, end‐diastolic left ventricular (LV) wall thicknesses, LV internal diameters, LV systolic function, left atrial size, or systolic and diastolic Doppler variables. One control cat developed hypertension. One high‐salt group cat developed persistent azotemia. Serial plasma biochemistry and urine specific gravity suggested early chronic kidney disease in 4 nonazotemic cats (2 per group), consistent with necropsy findings.

**Conclusions and Clinical Importance:**

In healthy aged cats, a commercial veterinary diet containing 3.26 ± 0.30 g/Mcal ME sodium was safe with regard to renal and cardiac function for up to 5 years.

**Abbreviations**
2D: 2‐dimensional
Ao: aorta
BUN: blood urea nitrogen
C: control
CKD: chronic kidney disease
E : A: early to late diastolic velocity ratio
FS: fractional shortening
GFR: glomerular filtration rate
Hbg: hemoglobin
HR: heart rate
HS: high salt
Htc: hematocrit
IQR: interquartile range
IVRT: isovolumic relaxation time
IVS: interventricular septum
IVSd: interventricular septum thickness at end‐diastole
IVSs: interventricular septum thickness at end‐systole
LA: left atrium
LIA: left interlobar artery
LMMs: linear mixed models
LRA: left renal artery
LV: left ventricular
LVDd: left ventricular diameter at end‐diastole
LVDs: left ventricular diameter at end‐systole
LVFW: left ventricular free wall
LVFWd: left ventricular free wall thickness at end‐diastole
LVFWs: left ventricular free wall at end‐systole
ME: metabolizable energy
MVG: myocardial velocity gradient
NRC: National Research Council
RI: resistive index
RIA: right interlobar artery
RRA: right renal artery
SABP: systolic arterial blood pressure
TDI: tissue Doppler imaging
UPC: urine protein : creatinine ratio
USG: urine specific gravity

## 1 INTRODUCTION

Feeding cats diets with a higher sodium chloride concentration than standard dry diets is an effective way to increase water consumption, resulting in urine dilution.^1^, ^2^ Some commercial veterinary diets for urolithiasis management and prevention are formulated with increased sodium content.^3^, ^4^, ^5^

In humans, high salt intake is associated with increased blood pressure and systemic arterial hypertension,^6^, ^7^, ^8^ which are the risk factors for major cardiovascular events including stroke and myocardial infarction,^6^, ^9^, ^10^ and incident chronic kidney disease (CKD).^11^

These associations have prompted studies in cats to investigate the cardiovascular and renal effects of high‐salt veterinary diets. For example, the level of sodium chloride intake did not affect systolic blood pressure in healthy adult cats,^12^, ^13^ healthy aged cats,^12^, ^14^ or cats with experimentally induced renal impairment.^15^ Renal function was not adversely affected by high salt intake for 7 days in either healthy cats or cats with renal impairment.^15^ In a crossover study comparing a high‐salt with a low‐salt diet, 6 cats with nonazotemic CKD had increases in blood urea nitrogen, serum phosphorus, and serum creatinine concentrations when fed the high‐salt diet. This outcome appeared to be a physiologic response rather than an indication of diet‐induced kidney injury and was at least in part reversed when the low‐salt diet subsequently was fed.^12^

Despite evidence from multiple studies that high‐salt diets are not detrimental to cardiovascular or renal health in cats, longer‐term studies are needed, especially in healthy aged cats, because of the positive association between age and blood pressure^16^, ^17^, ^18^ and the decrease in renal function with age.^19^

The objective of our prospective, randomized, blinded, and controlled study was to evaluate the renal and cardiovascular safety of a high‐salt diet fed to healthy aged cats for 5 years. Results from the first 2 years of the study have been published earlier.^20^, ^21^

## 2 MATERIALS AND METHODS

Full methodological details are published with the 2‐year results.^20^, ^21^

### 2.1 Study population and husbandry

**FIGURE 1:**
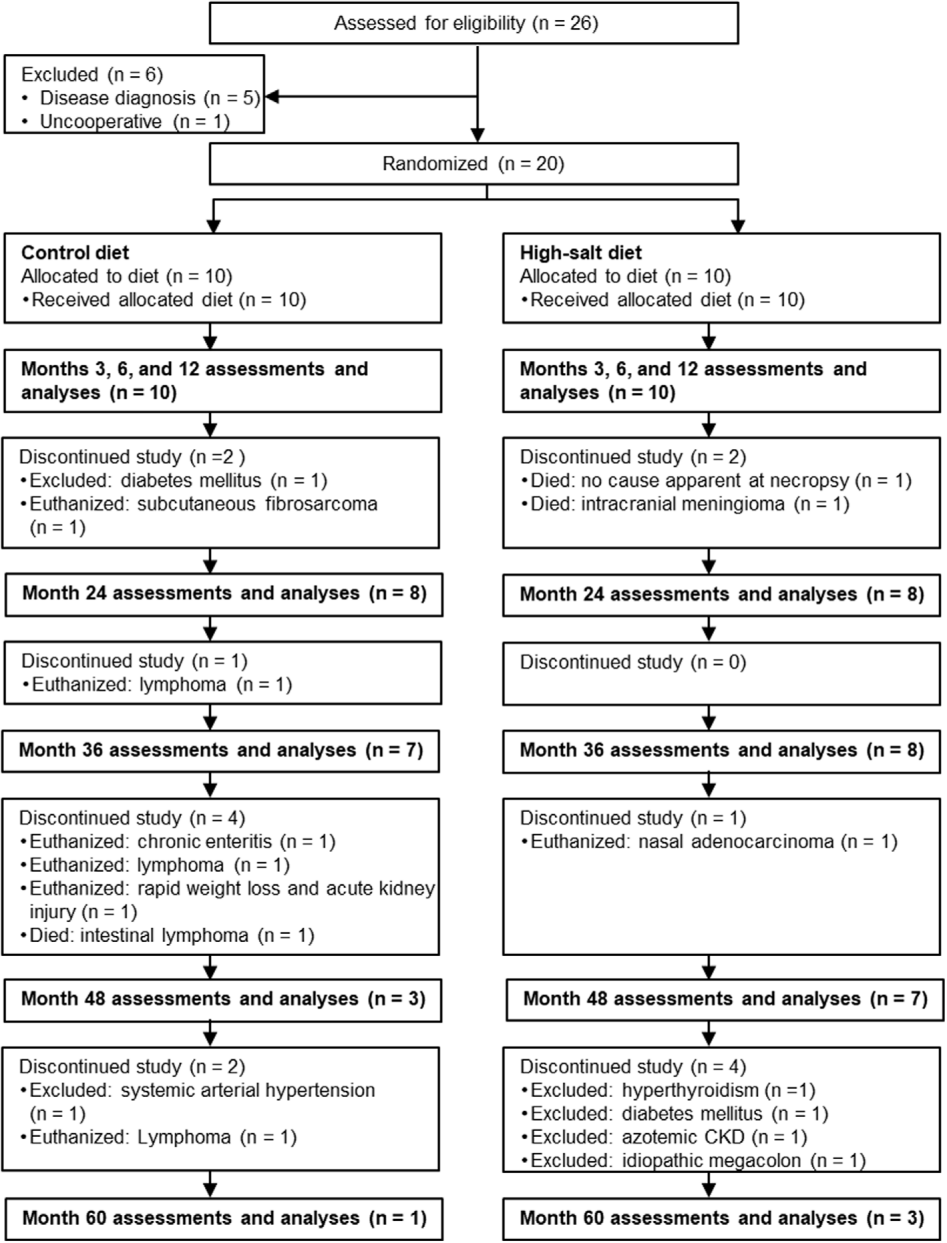
Flow chart of cats through the 60‐month study period. Euthanasia was not undertaken for the purposes of research; it was only permitted on compassionate grounds when medical treatment for a health condition was inappropriate or unsuccessful in maintaining the quality of life. CKD, chronic kidney disease.

Twenty‐six aged, neutered, domestic shorthair research colony cats were screened for eligibility (Figure 1). At baseline, cats had to be healthy based on standard veterinary evaluations, kidney ultrasonography, standard echocardiography, and conventional cardiac Doppler examination and had to be temperamentally cooperative.

Cats were housed in their randomized groups in an indoor research facility (Unité de Nutrition et d'Endocrinologie, Oniris, F‐44307, Nantes Cedex, France) with a 12‐h light/dark cycle, and controlled temperature (18°C‐21°C) and ventilation (250 m^3^/h, 12 h/d). From screening onward, cats were fed a maintenance dry expanded diet with a sodium content of 2.3 g/Mcal (Veterinary Cat Neutered, Young Male, Royal Canin SAS, Aimargues, France; Table 1), which was the same diet they had received for most of their life after neutering. Cats had free access to water.

**TABLE 1:** Nutrient composition of study diets.

Nutrient	Maintenance diet before study entry g/Mcal ME	Control diet g/Mcal ME	High‐sodium diet g/Mcal ME
Moisture	17.1	15.1 ± 1.6	14.0 ± 1.1
Protein	112.0	84.4 ± 3.2	86.8 ± 3.5
Fat	31.0	38.6 ± 1.2	38.3 ± 2.0
Dietary fiber	36.0	18.2 ± 2.1	16.2 ± 1.96
Minerals	23.7	14.9 ± 0.4	20.9 ± 1.2
Calcium	3.6	1.86 ± 0.23	1.89 ± 0.25
Total phosphorus	3.5	2.18 ± 0.18	2.18 ± 0.13
Inorganic phosphorus	1.0	1.00	1.00
Calcium: phosphorus ratio	1.03	0.85	0.87
Magnesium	0.200	0.125	0.125
Sodium	2.3	1.02 ± 0.16	3.26 ± 0.30
Chloride	4.0	2.26 ± 0.33	5.71 ± 0.28
Potassium	2.40	2.50	2.50
Energy, kcal/kg, NRC 2006	3530	4050 ± 131	3972 ± 79
Ingredients	Rice, poultry and poultry by‐product meal, wheat gluten, corn flour, poultry fat, corn gluten, minerals (including salt), cellulose, hydrolyzed animal protein (aroma), fish oil, fructo‐oligosaccharides, soy oil, vitamins, egg powder, dl‐methionine, hydrolyzed crustaceans (source of glucosamine), taurine, and marigold		

*Note*: Values are mean ± SD from 6 batches of control and 8 batches of high‐salt diet used over the 5 years of the study. Values are slightly different from the 2‐year report as more batches were produced. Inorganic phosphorus, magnesium, and potassium were calculated from the diet formulation rather than measured directly. Inorganic phosphorus (0.5 g/Mcal) was from Na pyrophosphate and 0.5 g/Mcal was from potassium monophosphate. The prior maintenance diet was Vet Cat Neutered, Young Male, Royal Canin SAS, Aimargues, France (typical analysis). The high‐sodium diet was Feline Veterinary Diet Urinary High Dilution. Royal Canin SAS, Aimargues, France. The same formula was used to produce both diets except that in the control diet formula most of the salt was replaced by corn flour.

Abbreviations: ME, metabolizable energy; NRC, National Research Council.

### 2.2 Study design

Our study was a prospective, randomized, blinded, and controlled trial of a high‐salt diet for up to 60 months. The protocol was reviewed and approved by the animal care and use committee of “Pays‐de‐la‐Loire” (file CEEA.2012.152) and by Royal Canin's ethics committee. The study complied with conditions approved by the French Ministry of Agriculture and the guidelines of the Guide for Care and Use of Laboratory Animals.^22^

Eligible cats were stratified into 3 subsets based on cardiac 2‐dimensional (2D) color tissue Doppler imaging (TDI) at baseline (normal, subnormal, and abnormal).^20^ Subnormality indicated the presence of regional postsystolic contraction waves for the left ventricular (LV) free wall (LVFW), the interventricular septum (IVS), or both without any other alteration. Abnormality indicated mild to moderate regional diastolic alterations characterized by an early to late diastolic velocity ratio (E : A ratio) < 1.^23^ In each subset, cats were ranked and paired based on glomerular filtration rate (GFR). Cats in each pair were randomized to the control or high‐salt diet by coin flip. Blinding to diet allocation is detailed in the Data S1.

The high‐salt (test) diet contained 3.26 ± 0.30 g/Mcal metabolizable energy (ME; mean, SD) of sodium (1.3% as fed) and 5.71 ± 0.28 g/Mcal ME of chloride (2.27% as fed; Table 1). The control diet had a sodium and chloride content of 1.02 ± 0.16 g/Mcal ME (0.35%) and 2.26 ± 0.33 g/Mcal ME (0.70%), respectively (Table 1). Cats had individual access via programmed collars to 70 g/day of their allocated diet, equivalent to the energy requirements of a 4.8 kg cat in good body condition (100 × bw^0.67^).

In the 2‐year study reported earlier, we included 3‐month and 6‐month follow‐ups; in the 5‐year study, we elected to report only the yearly follow‐ups.^20^, ^21^ Procedures and analyses detailed below were conducted at baseline and months 12, 24, 36, 48, and 60, except for body weight measured weekly and food consumption measured daily.

Cats that completed the study remained on their randomized diet and received standard veterinary care.

### 2.3 Body condition score

Monthly body condition scoring was planned with a 5‐point scale: 1 = emaciated or very thin, 3 = ideal, and 5 = obese or grossly obese.^24^ This measurement was mistakenly neglected for the first 6 months.

### 2.4 Hematology, blood biochemistry, and hormone assay

Blood samples were taken by jugular venipuncture and cephalic vein microsampling.^25^ Plasma biochemistry was performed using dry‐slide technology (Vitros 250 Chemistry System, Ortho‐Clinical Diagnostics, Raritan, New Jersey). The measurements included albumin, bilirubin, glucose, cholesterol, triglycerides, sodium, potassium, chloride, total carbon dioxide, calcium, phosphate, total protein, urea and creatinine concentrations, and alanine aminotransferase and alkaline phosphatase activities.^26^, ^27^, ^28^ Serum total thyroxine concentration was measured using a validated chemiluminescent immunoassay (Chemiluminescent Immulite 2000, DPC, Los Angeles, California).^26^, ^27^, ^28^

### 2.5 Urinalyses

Urine samples were collected by cystocentesis. Spot urinalysis included urine specific gravity (USG) measured by medical refractometer (Atago, Fisher Bioblock, Illkirch, France) and urine protein/creatinine ratio (UPC).^5^, ^29^

### 2.6 Glomerular filtration rate

Glomerular filtration rate was determined from the plasma clearance of a nominal dose of 20 mg/kg exogenous creatinine.^21^ Creatinine concentrations before and 5 minutes, 30 minutes, and 1, 2, 3, 5, and 8 hours after an IV bolus of creatinine (anhydrous creatinine, Sigma Chemical Co., St. Louis, Missouri) were analyzed using noncompartmental methods (WinNonlin version 5.2 Pharsight, Mountain View, California). Glomerular filtration rate was calculated as the creatinine dose divided by the area under the creatinine concentration versus time curve; unless otherwise stated, GFR was weight adjusted.

### 2.7 Blood pressure

Systolic arterial blood pressure (SABP) was measured indirectly in awake cats by the same trained operators using a Doppler system (811‐BL Parks Medical Electronics Inc, ALOHA, Oregon).^30^, ^31^ Systemic arterial hypertension was defined as an average SABP (from 5 readings) >160 mmHg.^21^, ^31^

### 2.8 Renal ultrasonography

A single experienced veterinary echographer (IT) blinded to the diet assignment measured the ultrasonographic aspect, length, width, and height of both kidneys. Resistive indices of renal and interlobar arteries were measured from a longitudinal section of each kidney by Doppler examination using an ultrasonographic unit with a microconvex 8 MHz probe (Biosound MyLab30 Universal Medical Systems Inc, Bedford Hills, New York).^21^ The resistive index was calculated as (peak systolic velocity − end‐diastolic velocity) divided by peak systolic velocity.

### 2.9 Standard echocardiography and conventional Doppler examination

Standard transthoracic echocardiography and conventional Doppler examinations were performed by an experienced board‐certified cardiologist (VC) blinded to diet assignment, in awake standing cats, with continuous ECG monitoring, using an ultrasonographic unit (Vivid 7 Dimension, General Electric Medical System, Waukesha, Wisconsin) equipped with 2 phased‐array transducers (4‐8 MHz and 4.5‐11.5 MHz). All procedures and methodologies have been validated earlier.^20^, ^30^, ^32^, ^33^ End‐diastolic and end‐systolic LV internal diameters, LVFW, and IVS thicknesses were measured from the right parasternal short‐axis view with 2D‐guided M‐mode echocardiography.^30^, ^34^ Two‐dimensional measurements included aortic (Ao) and left atrial (LA) diameters at end‐diastole and the end‐diastolic subaortic IVS thickness from the right parasternal 5‐chamber view at the level of the attachment of the left chordae tendineae to the mitral valve leaflets.^23^, ^30^ Pulsed wave Doppler mode variables were peak systolic aortic flow velocity and peak early and late diastolic mitral flow velocities (mitral E and A waves, respectively) from the left apical 5‐ and 4‐chamber views, respectively, and isovolumic relaxation time (IVRT), defined as the time interval between the end of aortic flow velocity and the onset of transmitral flow, taken from the left apical 5‐chamber view. Left atrial enlargement was defined as end‐diastolic LA:Ao >1.2^25^ and LV enlargement as internal LV diameter > the 95% prediction interval assessed according to body weight from a large population of healthy cats, with LV shortening fraction considered normal if within the corresponding 95% prediction interval.^35^ The hypertrophic myocardial phenotype was defined as at least 1 2D or M‐mode end‐diastolic LV myocardial wall thickness ≥6 mm.^36^

### 2.10 Two‐dimensional color tissue Doppler imaging

Examinations by 2D color tissue doppler imaging (TDI) were undertaken by the same operator blinded to the diet assignment using the same ultrasonographic unit as for standard echocardiography in awake standing cats with continuous ECG monitoring. Digital images were analyzed with specialist software (Echopac Dimension, General Electric Medical System, Waukesha, Wisconsin). Full details are published elsewhere.^20^, ^23^ In brief, peak myocardial velocities resulting from radial LVFW motion were measured in systole and in early and late diastole (S, E and A waves, respectively) in the subendocardial and subepicardial LVFW segments from the right parasternal ventricular short‐axis view.^20^ Peak systolic and early and late diastolic longitudinal velocities were measured with the standard left apical 4‐chamber view in 3 myocardial segments (2 from the LVFW at the base and the apex and 1 from the IVS at the base).^20^ The TDI diastolic E:A ratio was calculated for each of these 5 myocardial segments. Radial systolic myocardial velocity gradient (MVG) was the difference between subendocardial and subepicardial systolic velocities, and longitudinal systolic MVG was the difference between basal and apical systolic LVFW velocities. Mean heart rate was calculated from ECG monitoring during each radial and longitudinal TDI examination.

### 2.11 Necropsy examinations

Necropsy examinations, including histopathology, were conducted (JA, LD) on cats that died naturally or were euthanized during the study because of any severe disease resulting in unacceptably poor quality of life.

### 2.12 Retrospective case review of renal function

After the study end, an expert veterinary nephrologist (JE) blinded to the diet assignment and necropsy findings evaluated serial results of plasma creatinine and urea concentrations, spot USG, and body weight to identify cats with evidence of deteriorating renal function.

### 2.13 Statistical analysis

Two groups of 10 cats powered the study to detect a 20% change in GFR and blood pressure. There was 85% power to detect effect sizes of 0.4 ± 0.3 mL/min/kg for GFR and 20 ± 15 mmHg for SABP.

Statistical analyses were performed with R Core Team 2022. Descriptive statistics were expressed as median (interquartile range [IQR]) unless otherwise stated. Glomerular filtration rate, blood creatinine concentrations, and SABP, echocardiographic, Doppler, and TDI variables were analyzed for changes over time using linear mixed models (LMMs). Log or rank transformation was used as appropriate to meet statistical assumptions of LMMs (normally distributed residuals and homoscedasticity). Diet, time, and their interaction were fixed effects, and animals were a random effect; time was treated as a continuous variable. The LMMs for body weight (mean per cat for each year) had an additional fixed effect of sex. Effect sizes for GFR, blood creatinine concentrations, SABP, and imaging variables were calculated by pairwise comparisons at each time point with Cohen's *d* effect size for nontransformed or log‐transformed variables, and *r* Rosenthal's effect size for rank‐transformed variables. *P* ≤ .05 was considered significant.

Kaplan‐Meier analysis was performed on events of study discontinuation, such as meeting exclusion criteria, death, or euthanasia for a severe condition. Study survival (ie, ongoing continuation in the study) was compared between diet groups using a log‐rank test. The disease frequencies were compared between groups using the Chi‐squared test.

## 3 RESULTS

### 3.1 Study population

Twenty healthy neutered cats were enrolled, and 5 male and 5 female cats were randomized to each diet (Figure 1). Groups were similar at baseline for all study variables (Tables 2, 3, and S1‐S4). Age and body weight were (median ± [IQR]) 11.0 years (8.0‐11.6) and 5.06 kg (4.32‐5.64), respectively, for the control group and 11.6 years (11.5‐11.6) and 4.51 kg (3.90‐4.98), respectively, for the test group. Baseline 2D color TDI was normal for 8 cats, subnormal for 6 cats, and abnormal for 6 cats.^20^, ^23^ Baseline GFR was 1.9 mL/min/kg (1.8‐2.1) in the control group and 1.9 mL/min/kg (1.7‐2.1) in the test group.

**TABLE 2:** GFR, plasma creatinine concentration, spot urine analyses, and systolic arterial blood pressure in aged cats fed a control or high‐salt diet for up to 60 months.

Variable [reference interval]	Month 0		Month 12		Month 24		Month 36	
	C, n = 10	HS, n = 10	C, n = 10	HS, n = 10	C, n = 8	HS, n = 8	C, n = 7	HS, n = 8
GFR, mL/min/kg [1.2‐4.9]	1.9 (1.8 to 2.1)	1.9 (1.7 to 2.1)	2.0 (1.8 to 2.3)	1.7 (1.4 to 1.9)	1.7 (1.6 to 2.0)	1.7 (1.4 to 1.8)	1.9 (1.5 to 2.1)	1.8 (1.5 to 2.1)
Creatinine mg/dL [1.01‐2.35]	1.40 (1.34 to 1.45)	1.50 (1.31 to 1.60)	1.58 (1.47 to 1.72)	1.60 (1.51 to 1.82)	1.31 (1.26 to 1.46)	1.45 (1.31 to 1.59)	1.25 (1.06 to 1.43)	1.29 (1.18 to 1.39)
Creatinine μmol/L [89.0‐207.0]	123.8 (118.4 to 128.4)	133.0 (115.5 to 141.6)	139.4 (129.9 to 151.7)	141.2 (133.3 to 160.8)	116.2 (111.4 to 129.1)	127.9 (116.1 to 140.3)	110.8 (93.6 to 126.6)	113.7 (104.7 to 122.6)
Spot USG [≥1.035]	1.044 (1.042 to 1.045)	1.044 (1.039 to 1.056)	1.043 (1.040 to 1.055)	1.034 (1.030 to 1.046)	1.034 (1.021 to 1.043)	1.031 (1.028 to 1.040)	1.018 (1.015 to 1.026)	1.026 (1.026 to 1.044)
Spot UPC [<0.2]	0.24 (0.20 to 0.30)	0.21 (0.19 to 0.25)	0.18 (0.16 to 0.22)	0.22 (0.17 to 0.26)	0.20 (0.18 to 0.20)	0.15 (0.1 to 0.2)	0.30 (0.30 to 0.30)	0.20 (0.10 to 0.30)
SABP, mm Hg [<160]	152 (145 to 153)	154 (151 to 156)	147 (133 to 154)	149 (142 to 152)	140 (138 to 147)	144 (135 to 149)	152 (143 to 157)	150^a^ (141 to 152)

Variable [reference interval]	Month 48		Month 60		Rate of change per month (time effect) (95% CI)		Difference between groups in rate of change (95% CI)
	C, n = 3	HS, n = 7	C, n = 1	HS, n = 3	C, n = 10	HS, n = 10	
GFR, mL/min/kg [1.2‐4.9]	1.7 (1.4 to 2.2)	1.7 (1.1 to 2.0)	1.2	1.3	−0.0056 (−0.013 to 0.0022); ***P* = .16**	−0.0022 (−0.0088 to 0.0044); ***P* = .50**	−0.0034 (−0.014 to 0.0068); ***P* = .51**
				1.5			
				0.9			
Creatinine mg/dL [1.01‐2.35]	1.24 (1.11 to 1.38)	1.56 (1.34 to 1.66)	1.43	1.19	−0.0039 (−0.0082 to 0.00038); ***P* = .07**	−0.0055 (−0.0091 to −0.0018); ***P* = .004**	0.0015 (−0.0041 to 0.0072); ***P* = .58**
				1.57			
				1.91			
Creatinine μmol/L [89.0‐207.0]	109.6 (98.6 to 122.3)	132.9 (110.7 to 145.5)	126.4	105.4	−0.348 (−0.729 to 0.0339); ***P* = .07**	−0.485 (−0.807 to −0.1635); ***P* = .004**	0.137 (−0.362 to 0.637); ***P* = .58**
				138.5			
				168.8			
Spot USG [≥1.035]	1.029 (1.022 to 1.038)	1.017 (1.015 to 1.031)	1.019	1.038	−0.000329 (−0.000492 to − 0.000166); ***P* < .001**	−0.000442 (−0.000579 to −0.000305); ***P* < .001**	0.000113 (−0.0000999 to 0.000326); ***P* = .29**
				1.030			
				1.013			
Spot UPC [<0.2]	0.20 (0.20 to 0.25)	0.10 (0.10 to 0.12)	0.40	0.20	−0.000178 (−0.00189 to 0.00153); ***P* =. 84**	−0.000778 (−0.00223 to 0.00067); ***P* = .29**	0.0006 (−0.00164 to 0.00284); ***P* = .59**
				0.10			
				0.5			
SABP, mm Hg [<160]	148^a^ (142 to 169)	150^b^ (143 to 157)	146	157	0.066 (−0.17 to 0.30); ***P* = .58**	−0.019 (−0.22 to 0.18); ***P* = .85**	0.084 (−0.23 to 0.40); ***P* = .59**
				149			
				162			

*Note*: Data are median (interquartile range) except for data at month 60, which are the values for individual cats. The rate of change per month is the slope estimate from linear mixed models with time between month 0 and month 48 as a continuous variable. The difference between groups is the slope estimate contrast (control minus high‐salt group). *P* ≤ .05 is significant.

Abbreviations: C, control group; CI, confidence interval; GFR, glomerular filtration rate; HS, high‐salt group; SABP, systolic arterial blood pressure; USG, urine specific gravity; UPC, urine protein : creatinine ratio.

^a^ One cat had SABP >160 mmHg.

^b^ Two cats had SABP >160 mmHg.

**TABLE 3:** Echocardiographic and standard Doppler variables in healthy aged cats fed a control or high‐salt diet for up to 60 months.

Variable	Month 0		Month 12		Month 24		Month 36	
	C (n = 10)	HS (n = 10)	C (n = 10)	HS (n = 10)	C (n = 8)	HS (n = 8)	C (n = 7)	HS (n = 8)
M‐mode variables								
LVDd, mm	14.4 (13.6 to 15.4)	13.4 (13.1 to 14.6)	15.2 (14.4 to 15.8)	14.1 (13.5 to 14.7)	13.7 (13.0 to 14.7)	13.8 (12.8 to 14.2)	14.8 (13.9 to 15.8)	13.6 (13.1 to 14.9)
LVDs, mm	6.5 (6.3 to 7.1)	6.8 (5.8 to 7.7)	6.5 (6.0 to 7.9)	6.1 (5.1 to 6.8)	5.9 (5.4 to 6.0)	5.8 (5.5 to 6.3)	6.1 (5.5 to 6.8)	6.1 (5.3 to 6.7)
LVFWd, mm	4.6 (4.3 to 4.9)	4.4 (4.2 to 4.5	4.4 (4.3 to 4.7)	4.4 (4.2 to 4.7)	4.5 (4.3 to 4.8)	4.1 (4.0 to 4.5)	4.5 (4.5 to 4.7)	4.1 (3.8 to 4.3)
LVFWs, mm	7.9 (7.3 to 8.5)	7.7 (7.1 to 7.9)	8.2 (7.8 to 8.5)	7.9 (7.6 to 8.6)	8.6 (8.3 to 8.9)	7.9 (7.4 to 8.5)	8.4 (8.2 to 8.8)	7.7 (7.2 to 8.0)
IVSd, mm	4.8 (4.5 to 5.1)	4.9 (4.5 to 5.1)	4.7 (4.4 to 5.0)	4.7 (4.4 to 5.1)	4.6 (4.4 to 4.8)	4.3 (4.2 to 4.4)	4.6 (4.3 to 4.7)	4.2 (3.9 to 4.5)
IVSs	7.6 (7.0 to 8.2)	7.7 (7.1 to 8.0)	8.1 (7.6 to 8.5)	8.0 (7.8 to 8.3)	8.3 (7.9 to 8.7)	7.8 (7.4 to 8.0)	8.4 (7.9 to 8.6)	8.0 (6.7 to 8.3)
FS, %	53.5 (52.0 to 59.0)	51.0 (45.8 to 57.0)	56.5 (49.8 to 60.0)	57.5 (55.0 to 59.8)	60.0 (53.3 to 62.3)	57.0 (53.8 60.3)	59.0 (55.1 to 62.0)	56.1 (53.2 to 60.8)
Two‐dimensional variables								
LA:Ao	0.84 (0.77 to 0.91)	0.85 (0.79 to 0.85)	0.84 (0.77 to 0.92)	0.80 (0.74 to 0.92)	0.76 (0.72 to 0.83)	0.76 (0.66 to 0.82)	0.77 (0.70 to 0.84)	0.81 (0.75 to 0.83)
Subaortic IVSd, mm	4.3 (4.1 to 4.7)	4.4 (4.1 to 4.9)	4.4 (4.3 to 4.7)	4.3 (4.1 to 4.8)	4.4 (4.1 to 5.0)	4.4 (4.2 to 4.6)	4.2 (4.1 to 4.8)	4.2 (4.1 to 4.5)
Doppler variables								
Peak aortic flow velocity, m/s	1.2 (1.0 to 1.2)	1.2 (1.0 to 1.3)	1.2 (1.1 to 1.3)	1.2 (1.1 to 1.4)	1.0 (0.9 to 1.0)	1.1 (1.2 to 1.2)	1.0 (1.0 to 1.2)	1.0 (1.0 to 1.2)
Mitral E:A ratio	1.42 (1.30 to 1.72)	1.19 (1.07 to 1.38)	1.27 (1.20 to 1.33)	1.19 (1.11 to 1.32)	1.22 (1.14 to 1.32)	1.26 (1.06 to 1.51)	1.14 (1.04 to 1.53)	1.06 (0.77 to 1.09)
IVRT, ms	49 (45 to 53)	48 (39 to 58)	47 (43 to 51)	50 (48 to 53)	52 (44 to 56)	52 (47 to 55)	49 (47 to 53)	48 (46 to 49)

Variable	Month 48		Month 60		Rate of change per month (time effect) (95% CI)		Difference between groups in rate of change (95% CI)
	C (n = 3)	HS (n = 7)	C (n = 1)	HS (n = 3)	C (n = 10)	HS (n = 10)	
M‐mode variables							
LVDd, mm	15.5 (13.7 to 16.0)	14.5 (14.1 to 14.8)	14.3	13.3	0.0018 (−0.021 to 0.025); ***P* = .88**	0.010 (−0.0092 to 0.029); ***P*** = **.30**	−0.0083 (−0.038 to 0.022); ***P*** = **.58**
				12.5			
				14.7			
LVDs, mm	7.6 (7.0 to 8.0)	5.9 (5.5 to 6.9)	7.7	6.1	−0.015 (−0.036 to 0.062); ***P* = .16**	−0.0090 (−0.027 to 0.0087); ***P* = .31**	−0.0058 (−0.033 to 0.022); ***P* = .68**
				4.5			
				5.9			
LVFWd, mm	4.5 (4.3 to 4.7)	4.4 (4.1 to 4.5)	4.4	3.9	−0.0018 (−0.0075 to 0.0039); ***P* = .53**	−0.0048 (−0.096 to 0.000013); ***P* = .05**	0.0030 (−0.0045 to 0.011); ***P* = .43**
				4.2			
				4.3			
LVFWs, mm	8.4 (8.3 to 8.7)	7.7 (7.6 to 7.9)	7.1	7.1	0.020 (0.0074 to 0.032); ***P* = .002**	0.00029 (−0.010 to 0.011); ***P* = .96**	0.020 (0.0033 to 0.036); ***P* = .02**
				7.7			
				8.2			
IVSd, mm	4.5 (4.3 to 4.6)	4.3 (4.1 to 4.6)	4.5	3.8	−0.0085 (−0.015 to −0.0018); ***P* = .01**	−0.013 (−0.019 to −0.0075); ***P* < .001**	0.0046 (−0.0041 to 0.013); ***P* = .30**
				4.2			
				4.4			
IVSs	7.6 (7.2 to 8.0)	7.7 (7.2 to 8.1)	7.7	7.1	0.012 (0.0014 to 0.023); ***P* = .03**	−0.0025 (−0.012 to 0.0066); ***P* = .58**	0.015 (0.00063 to 0.029); ***P* = .04**
				7.6			
				8.5			
FS, %	50.0 (48.0 to 50.5)	61.0 (54.0 to 62.5)	46	54	0.11 (0.0048 to 0.22); ***P* = .04**	0.10 (0.011 to 0.19); ***P* = .03**	0.011 (−0.13 to 0.15); ***P* = .87**
				64			
				60			
Two‐dimensional variables							
LA:Ao	0.78 (0.76 to 0.79)	0.81 (0.76 to 0.92)	0.61	0.87	−0.0021 (−0.0042 to 0.00012); ***P* = .06**	−0.00040 (−0.0023 to 0.0014); ***P* = .66**	−0.0017 (−0.0045 to 0.0012); ***P* = .25**
				0.69			
				0.74			
Subaortic IVSd, mm	4.1 (3.7 to 4.4)	4.1 (4.1 to 4.5)	4.1	4.1	−0.0012 (−0.0079 to 0.0056); ***P* = .73**	−0.0056 (−0.011 to 0.00010); ***P* = .05**	0.0044 (−0.0044 to 0.013); ***P* = .32**
				3.9			
				4.5			
Doppler variables							
Peak aortic flow velocity, m/s	1.0 (0.9 to 1.1)	1.2 (1.1 to 1.2)	1.0	1.3	−0.0030 (−0.0063 to 0.00028); ***P* = .07**	−0.0018 (−0.0045 to 0.0010); ***P* = .21**	−0.0013 (−0.0055 to 0.0030); ***P* = .56**
				1.0			
				1.0			
Mitral E:A ratio	0.93 (0.78 to 1.08)	0.88 (0.67 to 1.21)	0.86	1.09	−0.010 (−0.017 to −0.0036); ***P* =** .**003**	−0.0076 (−0.013 to −0.0022); ***P* = .01**	−0.0025 (−0.011 to 0.0060); ***P* = .56**
				0.68			
				0.78			
IVRT, ms	48 (46 to 50)	48 (45 to 50)	49	48	0.039 (−0.11 to 0.18); ***P* = .59**	−0.048 (−0.17 to 0.077); ***P* = .45**	0.087 (−0.10 to 0.28); ***P* = .37**
				49			
				43			

*Note*: Data are median (interquartile range) except for data at month 60, which are the values for individual cats. The rate of change per month is the slope estimate from linear mixed models with time between month 0 and month 48 as a continuous variable. The difference between groups is the slope estimate contrast (control−high‐salt group). *P* ≤ .05 is significant.

Abbreviations: C, control‐diet group; CI, confidence interval; E, peak velocity of early diastolic transmitral flow; A, peak velocity of late diastolic transmitral flow; FS, fractional shortening; HS, high‐salt diet group; IVRT, isovolumic relaxation time assessed by pulsed wave Doppler; IVSd, interventricular septum thickness at end‐diastole; IVSs, interventricular septum thickness at end‐systole; LA : Ao, end‐diastolic left atrium : aorta ratio; LVDd, left ventricular diameter at end‐diastole; LVDs, left ventricular diameter at end‐systole; LVFWd, left ventricular free wall thickness at end‐diastole; LVFWs, left ventricular free wall at end‐systole.

### 3.2 Study survival and discontinuations

**FIGURE 2:**
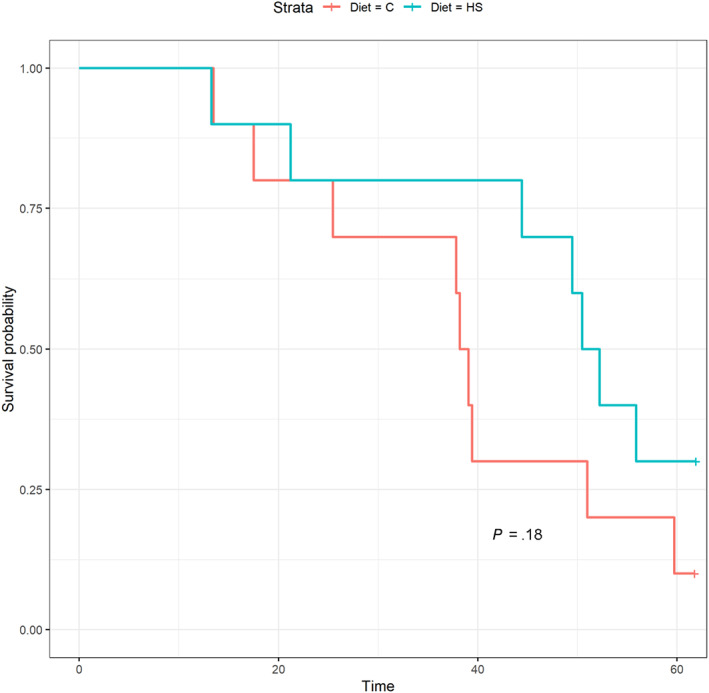
Kaplan‐Meier survival analysis for continuation in the study. There was no significant difference between diet groups in the probability of remaining in the study (*P* = .18). C, control; HS, high salt.

Figure 1 summarizes study discontinuations. No significant difference was found between diet groups in the probability of remaining in the study (Figure 2, *P* = .18). Time in the study was 38.7 months (28.6‐48.2) for the control group and 51.4 months (45.7‐59.0) for the test group. All other statistical analyses excluded data at month 60 because only 4 cats remained in the study.

Necropsy examinations were performed on 6 of 7 cats euthanized during the study because of severe disease, 3 cats that died naturally, 4 cats that completed the 60‐month study and were later euthanized, and 1 of 6 cats that were excluded during the study (Figure 1 and Table S5). No cats were euthanized for, or died from, cardiovascular disease or CKD during the study. Two cats were removed from the study because of a cardiovascular or renal disorder: a control cat (case 8; 15.1 years old) excluded after 51.0 months because of persistent systemic arterial hypertension and 1 cat in the test group (case 12; 15.9 years old) that was excluded after 50.5 months because of azotemia (plasma creatinine concentration >180 μmol/L [>2 mg/dL]). The case review found deteriorating renal function indicative of early CKD in 4 nonazotemic cats (2 in each diet group [cases 7, 17, 19, and 25]), which was supported by necropsy findings of CKD. Of these 4 cats, 2 completed the 60‐month study (1 control cat euthanized 1.9 months after study end and 1 cat in the test group euthanized 3.6 months after study end, both for CKD), 1 control cat died from lymphoma, and 1 cat in the test group was excluded for megacolon and euthanized 4.4 months later. One cat in the test group (case 13) had no biochemical evidence of renal deterioration during the study but was euthanized 3.5 years after study end with CKD complicated by pneumonia.

### 3.3 Hematology, plasma biochemistry, and serum thyroxine

Hematologic variables were mainly within the reference intervals (Table S1). Platelet counts often were slightly lower than reference levels with no apparent pattern, except for 1 cat excluded after month 24 with lymphoma (case 4) and another considered to have early CKD (case 19).

Biochemistry variables also were mainly within reference intervals (Table S2). Median sodium concentrations were slightly below the reference interval at months 24 and 36 in the control group and at months 0, 24, 48, and 60 in the test group.

Serum total thyroxine concentrations remained within the laboratory reference interval except for 1 cat in the test group that was diagnosed with hyperthyroidism (serum thyroxine, 7.7 μg/dL) and left the study after month 36.

### 3.4 Indicators of renal function

Descriptive statistics, rates of change, and effect sizes for renal function variables are presented in Tables 2 and S6. Glomerular filtration rates tended to decrease over time but no significant difference was found in rate of decrease between groups (*P* = .51) or in effect sizes except at month 12. In all cats, GFR was ≥1 mL/min/kg except 1 cat in the test group (case 25) that had GFR 0.9 mL/min/kg at month 60 and was diagnosed with CKD after the study end. This cat was diagnosed with early CKD in the case review (Table S5). The single cat that developed azotemia (case 12) and the 4 nonazotemic cats identified in the case review with early signs of CKD (cases 7, 17, 19, and 25) had final GFRs between 0.9 and 1.2 mL/kg/min. Of the 15 other cats, 14 had final GFR ≥1.5 mL/min/kg and 1 had GFR of 1.3 mL/min/kg at month 60 (case 13). No significant difference was found between the diet groups in the frequency of CKD (*P* = .99).

Plasma creatinine concentration decreased over time in the test group, but there was no significant difference compared to the control (*P* = .58; Table 2) or significant effect size at any time point (Table S6). The case review attributed trends in plasma creatinine and urea concentrations in individual cats to hyperthyroidism, CKD, or early CKD, except for 1 control cat (case 22) with unexplained decreasing USG (2 values <1.030).

Urine specific gravity values decreasing to <1.030 were apparent in cats diagnosed with CKD or considered to have early CKD in the case review (Table S5). Median UPC was slightly below or above 0.2 in both diet groups at all time points.

### 3.5 Blood pressure

No significant change in SABP over time was found in or between groups (*P* = .59; Table 2) or in effect sizes at any time point (Table S6). Median SABP for each group was <160 mmHg at all time points (Table 2). The single control cat (case 8) with SABP >160 mmHg (190 mmHg at month 48) was discontinued because of overt systemic hypertension with no biochemical evidence of renal dysfunction (no necropsy examination). In the test group, 3 cats had at least 1 SABP measurement >160 mmHg. One of these cats had SABP of 191 mmHg at month 48 and was diagnosed with hyperthyroidism with no evidence of coexisting renal disease (case 9, no necropsy examination). The second cat (case 13) had SABP of 162 mmHg at month 36, but no evidence of renal disease was found during the study, although GFR decreased at month 60 to 1.3 mL/min/kg (Table S5). When this cat was euthanized because of CKD and aspiration pneumonia 3.5 years after study completion (aged 15.5 years), necropsy examination identified cardiovascular abnormalities consistent with hypertension secondary to CKD. The third cat had SABP of 162 mmHg at months 48 and 60 and case review showed evidence of early CKD, verified by necropsy examination when the cat was euthanized because of CKD 3.6 months after study end. No significant difference was found between the diet groups in frequency of hypertension (*P* = .99).

### 3.6 Cardiac and renal imaging

All 2D and M‐mode echocardiographic variables (n = 9) remained within reference intervals throughout the study for all cats.^23^, ^35^ The end‐diastolic LVFW thickness tended to decrease over time, but the rate was not significant in or between groups (*P* = .43; Table 3), and effect sizes were not significant except at month 36 (Table S6). The end‐diastolic IVS thickness decreased significantly over time in both groups, but no significant difference was found between them in rate of change (*P* = .30) or significant effect size at any time point.

Similarly, rates of change of all other 2D and M‐mode variables were not significantly different between the 2 groups, except for end‐systolic LVFW thickness and end‐systole IVS thickness (Table 3). No systolic anterior mitral valve motion, leading to LV outflow tract obstruction, was detected in any cat using both 2D and M‐modes. No arrhythmias were detected by ECG.

Peak systolic aortic flow velocity and IVRT remained within reference intervals throughout the study for all cats, with no significant differences between groups in rates of change (Table 3).^20^, ^37^ The mitral E : A ratio decreased significantly over time in both groups with median values <1 at month 48, but the rate of change was not significantly different between them (*P* = .56), and effect sizes were nonsignificant (Table S6).

Several TDI E : A ratios decreased significantly (Table S3). In the test group, these were the E : A ratio for the radial LVFW motion measured in the subendocardial and subepicardial segments and the E : A ratio for the longitudinal IVS motion at the base. In the control group, significant decreases were observed in the E : A ratio for the LFVW longitudinal motion at the base and the S wave at the IVS base. The only rates of change that were significantly different between groups were the E : A ratio for the radial LVFW motion measured in the subepicardial segment (*P* = .01;effect sizes were not significant [Table S6]) and the longitudinal IVS S wave (*P* = .01;significant effect sizes at months 36 and 48). Descriptive statistics and rates of change are presented for renal resistive indices in Table S4. Rates of change were not significantly different between diet groups. Some individual results were above the reference interval (>72)^38^ at various time points in 5 control cats (including 1 cat at 2 time points) and 5 test cats (3 cats at 2 time points).

### 3.7 Food consumption, body weight, and body condition score

Food consumption decreased significantly over time in both groups, but the rate of change was not significantly different between them (−3.6 g/year [95% CI: −6.0 to −1.2, *P* = .001] for the control group and −1.2 g/year [−3.6 to −0.36, *P* = .05] for the test group; *P* = .12; Table 4). Body weight decreased significantly in both diet groups, but the rate of change was not significantly different between them (−93 g/year [−152 to −34, *P* = .003] vs −78 g/year [−124 to −32, *P* = .01]; *P* = .68; Table 4). Median body condition score (5‐point scale) (BCS) was 3/5 at months 12, 24, 36, and 48 in both diet groups except for month 48 when the control group had a median BCS of 2/5 (Table 4).

**TABLE 4:** Food consumption, bodyweight, and body condition score in healthy aged cats fed a control or high‐salt diet for up to 60 months.

Variable	Month 0		Month 12		Month 24		Month 36	
	C (n = 10)	HS (n = 10)	C (n = 10)	HS (n = 10)	C (n = 8)	HS (n = 8)	C (n = 7)	HS (n = 8)
Food intake^a^, g/d	NA^a^	NA	58.7 (56.8 to 62.2)	58.4 (52.7 to 60.8)	55.8 (54.7 to 59.2)	49.5 (43.9 to 56.7)	54.6 (47.9 to 57.0)	46.6 (45.7 to 51.5)
BW, kg	4.910 (4.460 to 5.465)	4.590 (3.870 to 5.025)	5.128 (4.400 to 5.728)	4.619 (4.016 to 5.236)	4.824 (4.067 to 5207)	4.203 (3.724 to 4.742)	4.650 (4.234 to 4764)	4.255 (3.545 to 4.623)
BCS^b^	3 (2 to 3)^b^	3 (2 to 3)^b^	3 (3 to 3)	3 (2 to 3)	3 (3 to 4)	3 (3 to 3)	3 (2 to 4)	3 (3 to 3)

Variable	Month 48		Month 60		Rate of change per month (time effect) (95% CI)		Difference between groups in rate of change (95% CI)
	C (n = 3)	HS (n = 7)	C (n = 1)	HS (n = 3)	C (n = 10)	HS (n = 10)	
Food intake^a^, g/d	52.7 (47.4 to 54.2)	46.5 (39.4 to 49.7)	35.0	43.4	−0.3 (−0.5 to −0.1); ***P* = .001**	−0.1 (−0.3 to −0.03); ***P* = .05**	−0.2 (−0.4 to 0.1); ***P* = .12**
				46.1			
				50.8			
BW, kg	3.987 (3.929 to 4.414)	4.302 (3.761‐4.635)	3.825	3.283	−7.77 (−12.7 to to 2.81); ***P* = .003**	−6.50 (−10.3 to −2.7); ***P* = .01**	−1.27 (−7.52 to −4.97); ***P* = .68**
				4.076			
				4.303			
BCS^b^	2 (2 to 2)	3 (2 to 3)	2	3	ND	ND	ND
				3			
				2			

*Note*: Data are median (interquartile range) except for data at month 60, which are the values for individual cats. The rate of change per month is the slope estimate from linear mixed models with time between month 0 and month 48 as a continuous variable. The difference between groups is the slope estimate contrast (control minus high‐salt group). *P* ≤ .05 is significant.

Abbreviations: BCS, body condition score (5‐point scale); BW, bodyweight; C, control‐diet group; CI, confidence interval; HS, high‐salt diet group; NA, not available; ND, not done.

^a^ Food intake is expressed as median gram/day over the preceding year, and therefore, there are no food intake data at month 0.

^b^ BCS was not recorded before month 6. The figures reported for month 0, therefore, correspond to month 6. Only descriptive statistics are presented for BCS because of the small sample size, the discrete nature of this variable, and the low variability.

## 4 DISCUSSION

We aimed to assess the safety of a high‐sodium diet in apparently healthy aged cats based on standard veterinary assessments. Chronic kidney disease and hypertension were of special interest because of their prevalence in aging cats and association with high salt intake in other species.^16^, ^19^, ^39^, ^40^, ^41^, ^42^, ^43^ Our findings support the long‐term safety of a high‐salt diet (sodium content 3.26 g/Mcal ME) in healthy cats at risk of renal and cardiovascular morbidities, using robust indicators of cardiac and renal function. Survival time in the study was not different between diet groups, and the causes of discontinuations were consistent with diseases associated with older cats, including CKD, lymphoma, diabetes mellitus, and hyperthyroidism. The occurrence of such diseases combined with early signs of renal disease detected in some cats at necropsy suggests that the study population, although small, was representative of a healthy aging population.

Glomerular filtration rate, the most sensitive measure of renal function, identified 2 groups of cats. One group consisted of cats diagnosed with CKD on necropsy examination or showing decreasing GFR (≤1.2 mL/min/kg) in the case review. The remaining cats maintained GFR ≥1.3 mL/min/kg (within the reference range)^44^ and mainly ≥1.5 mL/min/kg. The high‐salt diet had no effect on GFR over time, consistent with other studies in healthy older cats.^44^, ^45^

Over 5 years, 1 cat (5%) developed azotemia, a rate that approximates to the 5% estimated 2‐year prevalence of CKD in cats aged between 13.5 and 18 years in a United Kingdom primary care practice study.^40^ With the benefit of serial measurements of plasma creatinine concentration and USG over time, often not available in practice, the case review detected early CKD in 4 nonazotemic cats. A combined 25.0% rate of azotemic or early CKD over a median follow‐up of 39 months is consistent with a study of 216 healthy, client‐owned cats aged >9 years followed until death or development of azotemia.^46^ That study found an azotemia rate of 25.0% over 36 months and 29.6% over the entire study duration, which were probably underestimates because of the healthy cats lost to follow‐up.^46^ In another observational study of aged cats (median age, 13.0 years), 30.5% of cats developed azotemia over 12 months.^42^

Other than results in individual cats attributed to CKD, early CKD, hypertension (n = 1), and hyperthyroidism, there was no indication in any blood or urinary variables of consistent changes or adverse renal effects of the high‐salt diet. The high‐salt diet did not affect kidney perfusion relative to control diet as assessed by the resistive index for intrarenal blood flow.^47^, ^48^, ^49^ Values outside of reference intervals occurred with no apparent pattern and are expected with repeated measures over time.

Systolic blood pressure was not affected by the high‐salt diet, and there was no indication of primary hypertensive disease in the test group. These data support evidence from short‐term studies showing that increased dietary salt intake in healthy aged cats does not affect blood pressure.^12^, ^13^, ^14^, ^15^

The absence of clinically relevant long‐term cardiovascular effects of a high‐salt diet in healthy aged cats provides further evidence of species differences in physiological responses to high sodium intake. In humans, high salt intake is associated with fluid retention, increased arterial blood pressure, and hypertension,^6^, ^7^, ^8^, ^20^ and accentuates salt sensitivity with increasing age.^50^ Independent of blood pressure, high salt intake in humans also is associated with increased LV mass and LV wall thicknesses.^51^, ^52^, ^53^ In our study, the high‐salt diet did not induce diffuse or localized hypertrophic changes, LA or LV dilatation, systolic dysfunction, or LV outflow tract obstruction. Decreases in IVS thickness at end‐diastole were observed over time in both groups and might have been related to decreasing body weight.^37^ This observation combined with lack of significant change in LVFW thickness at end‐diastole does not support a deleterious effect of chronic high salt intake in aging cats.^53^, ^54^, ^55^ In humans and animal models, salt can directly trigger various arrhythmias,^54^, ^55^ mediated for example by stretch or increased intracellular calcium concentrations in cardiomyocytes. No arrythmias were detected in our study.

By measuring both global and regional myocardial velocities throughout the cardiac cycle, 2D color TDI is sensitive enough to detect early functional myocardial changes in the absence of myocardial hypertrophy, including those associated with systemic hypertension.^32^, ^33^ There were no apparent differences over time between diet groups in any variables of myocardial function measured by 2D color TDI except the E : A ratio measured in the subepicardial segment for the radial LVFW motion and the longitudinal IVS S wave. This finding suggests focal myocardial dysfunction, likely related to focal myocardial remodeling, such as myocardial fibrosis, with no impact on cardiac function.^56^

Excessive amounts of highly bioavailable dietary phosphorus (ie, inorganic phosphorus [Pi]) can lead to kidney damage in cats.^57^ Moderate amounts (1 g/Mcal Pi) over 7 months have been well tolerated in adult cats,^58^ but nutritional guidelines do not report a maximum safe level.^57^, ^59^ Although our study was not designed to evaluate the effect of Pi on renal health, kidney function was rigorously followed. During the study and for most of their lives, all cats had been fed diets containing 1 g/Mcal ME Pi (Table 1) in the form of potassium monophosphate (50%) and pyrophosphate (50%). The low frequency of azotemia (5%) during our study strongly suggests that 1 g/Mcal of dietary Pi (at least in the form of potassium monophosphate and pyrophosphate) is safe for chronic feeding. Potassium monophosphate has high bioavailability similar to sodium monophosphate.^60^

Body weight was stable in both diet groups for the first 2 years, after which it started to decrease. Some loss of body weight was expected with aging^61^ and would have been augmented as cats developed diseases associated with weight loss and left the study. The decrease in body weight was not detected by BCS, which remained relatively stable (mostly 2 or 3). The 5‐point BCS scale we used is less sensitive than the 9‐point scale commonly used now.^24^ The limitations of the 5‐point scale are evident when considering that an ideal BCS of 3 spans 3 scores in the 9‐point scale (ie, BCS of 4 [underweight], 5 [ideal], and 6 [overweight]). The starting median body weight of cats (5.1 kg in the control group and 4.5 kg in the test group) suggested that the BCS was underestimated relative to the 9‐point scale. Although feeding a fixed amount throughout the study could be perceived as a limitation, there was no indication that this factor was limiting in maintenance of body weight. Over the first 2 years, cats maintained their body weight, and overall, the rate of decrease in food consumption was not significantly different between groups. Although we measured body weight and BCS, they may not reflect muscle mass. Cats lost muscle mass, and the lack of longitudinal measurement of muscle condition score is a limitation.

Strengths of our study include its long duration and randomized, controlled, and blinded design. The diet groups were matched at randomization according to both cardiac and renal function, which was important because morbidity in 1 will affect function of the other.^30^, ^39^, ^62^ The risk of variability in imaging procedures was mitigated by using the same experienced operators throughout the study.^30^, ^32^ The ability to detect adverse effects of high salt intake in healthy cats was increased by studying a high‐risk population (aged cats) with a comprehensive range of renal and cardiac measurements, including GFR and 2D color TDI. The retrospective case review provided insights into the renal function of individual cats using variables that are routine in clinical practice. Necropsy examinations complemented clinical observations.

One limitation of the study was the small numbers of cats, particularly at the later time points. This concern was mitigated in part by analyzing rates of change in key safety variables. This was the most relevant statistic for a longitudinal study of aging animals, allowing data from cats leaving the study early to contribute to the overall evaluations. It was also important for distinguishing between age‐related and potential diet‐related changes. Decreases in body weight, GFR, and plasma creatinine concentration were expected with healthy aging regardless of diet. In secondary analyses, we calculated effect sizes for diet within time differences (Table S6) to estimate whether small sample sizes potentially were biasing statistical interpretation. For GFR, SABP, and plasma creatinine concentration, the predominance of small effect sizes and corresponding nonsignificant *P* values suggest that small sample size did not affect the overall results. In the test group, changes over time in LVFW thickness at end‐diastole did not reach significance (*P* < .05, Table 3), but a large effect size at 1 time point supports a difference in the rate of change between diet groups.

Minerals other than sodium and chloride can affect key variables of renal and cardiovascular health. In humans, potassium and magnesium are inversely related to blood pressure.^63^ The formulation‐based concentrations of potassium and magnesium were the same in both diets, and therefore, no confounding effect on cardiovascular health is expected.

In summary, chronic dietary sodium intake of 3.26 g/Mcal ME (~3‐fold higher than a standard diet) was safe with regard to renal and cardiac function, blood pressure, and other health outcomes in healthy aging cats. The frequency of CKD in study cats was generally lower than in observational population studies. In addition, a diet containing 1 g Pi/Mcal appeared to be safe in aged cats.

## CONFLICT OF INTEREST DECLARATION

J. Laxalde and V. Biourge are employees of Royal Canin SAS. J. Elliot has received several grants from Royal Canin to support the Royal Veterinary College feline kidney clinics in London and has been a paid consultant for Royal Canin and the Waltham Pet Science Institute. P. Nguyen's affiliated institute, the Nutrition and Endocrinology Unit at Oniris, received support from Royal Canin for a Research Convention. Hervé P. Lefebvre has received several grants from Royal Canin to support the development and validation of the exogenous creatinine GFR method. The protocol was designed collegially by all co‐authors except J. Laxalde and L. Dorso. All of the investigators were blinded to the treatment over the duration of the study. B. S. Reynolds, V. Chetboul, I. Testault, L. Dorso, and J. Abadie have no conflicts of interest to declare.

## OFF‐LABEL ANTIMICROBIAL DECLARATION

Authors declare no off‐label use of antimicrobials.

## INSTITUTIONAL ANIMAL CARE AND USE COMMITTEE (IACUC) OR OTHER APPROVAL DECLARATION

The protocol was reviewed and approved by the animal care and use committee of Pays‐de‐la‐Loire (file CEEA.2012.152) and by Royal Canin's ethics committee. The study complied with conditions approved by the French Ministry of Agriculture and the guidelines of the Guide for Care and Use of Laboratory Animals.

## HUMAN ETHICS APPROVAL DECLARATION

Authors declare human ethics approval was not needed for this study.

## Supporting information


**Data S1.** Supporting Information.

**Table S1.** Hematological variables in aged cats fed a control or high‐salt diet for up to 60 months.
**Table S2.** Plasma biochemistry in aged cats fed a control or high‐salt diet for up to 60 months.
**Table S3.** Two‐dimensional tissue Doppler imaging variables in aged cats fed a control or high‐salt diet.
**Table S4.** Renal resistive indexes in aged cats fed a control or high‐salt diet for up to 60 months.
**Table S5.** Case review of serial biochemistry and spot USG data, and key postmortem findings.
**Table S6.** Effect sizes for pairwise comparisons between diets at each time point.
