# Supplementary material for: Long‐term safety of dietary salt: A 5‐year ProspEctive rAndomized bliNded and controlled stUdy in healThy aged cats (PEANUT study)
Source: J Vet Intern Med. 2023 Dec 12;38(1):285–99. doi: 10.1111/jvim.16952 (PMC10800216; doi:10.1111/jvim.16952)
Supplement: Supplementary file 1 — Data S1. Supporting Information. [file JVIM-38-285-s001.pdf]

Supplementary Information

Procedures followed to maintain blindness related with treatment for the duration of the study.

The diets were delivered to the research facility in white bags with a code and the date of production. To avoid mistakes the kibble shape was round for one of the diets and triangular for the other. Feeding of the cats was managed by the animal technicians and their supervisor, who was the only one to know the diet codes. All but two investigators were based in other institutions and came to the facility to collect their samples and/or make their measurements. Cats were presented to them by the animal supervisor in a random order according to their diet. Samples sent for analysis and animals submitted for necropsy were only identified by the name and ID number. The statistician performed the analysis blindly; diets were only identified as diet A or diet B.

The diets were produced at a pilot facility and were subject to quality control measures. They were bagged in 10 kg neutral white bags impermeable to water and oxygen. An oxygen absorber was added to the bag to minimize oxidation. Prior studies demonstrated that these conditions preserve diets for at least 18 months, the main risk being oxidation.

Diets were produced at least once a year to ensure a constant supply. A representative sample of each batch was sent to an accredited laboratory for analysis (as reported in Table 1). Batches were shipped to the research facility after validation of their nutritional composition. No other analyses were performed after shipping.
