# Supplementary material for: Long‐term safety of dietary salt: A 5‐year ProspEctive rAndomized bliNded and controlled stUdy in healThy aged cats (PEANUT study)
Source: J Vet Intern Med. 2023 Dec 12;38(1):285–99. doi: 10.1111/jvim.16952 (PMC10800216; doi:10.1111/jvim.16952)
Supplement: Supplementary file 2 — Table S1. Hematological variables in aged cats fed a control or high‐salt diet for up to 60 months. Table S2. Plasma biochemistry in aged cats fed a control or high‐salt diet for up to 60 months. Table S3. Two‐dimensional tissue Doppler imaging variables in aged cats fed a control or high‐salt diet. Table S4. Renal resistive indexes in aged cats fed a control or high‐salt diet for up to 60 months. Table S5. Case review of serial biochemistry and spot USG data, and key postmortem findings. Table S6. Effect sizes for pairwise comparisons between diets at each time point. [file JVIM-38-285-s002.pdf]

Supplementary Table 1. Hematological variables in aged cats fed a control or high-salt diet for up to 60 months

Variable [reference interval]	Month 0		Month 12		Month 24		Month 36		Month 48		Month 60	
	C N = 10	HS N = 10	C N = 10	HS N = 10	C N = 8	HS N = 8	C N = 7	HS N = 8	C N = 3	HS N = 7	C N = 1	HS N = 3
**Hct% [24%-45%]**	41.9 (41.1;45.1)	38.6 (36.9;42.5)	33.2 (32.1;33.8)	28.5 (27.0;33.0)	35.4 (30.7;35.7)	35.1 (32.8;35.8)	28.6 (27.9;33.1)	32.3 (31.4;34.3)	35.7 (32.9;39.0	31.9 (28.2;35.7)	30.1 (30.1;30.1)	36.3 35.1 28.2
**Hgb, g/dL [8.0-15.0]**	13.1 (12.8;13.5)	12.2 (11.6;13.5)	9.9 (9.7;10.7)	9.0 (8.8;10.1)	11.0 (9.3;11.3)	10.7 (9.8;11.3)	9.0 (8.7;10.5)	10.1 (9.6;10.8)	10.9 (10.0;12.2)	10.0 (9.0;11.2)	9.1	11.8 10.5 8.9
**White blood cells, count/µL [5500-19500]**	10250 (9350; 11800)	10250 (8675; 11250)	11500 (9225; 13825)	10400 (9325; 12575)	11050 (10325; 17975)	13450 (10750; 14550)	11500 (10600; 17350)	11050 (10075; 12475)	9400 (7700; 10750)	9000 (8750; 10300)	10000	8500 16000 9600
**Platelets, count x 10^3/µL**	464 (422;593)	546 (530;569)	219 (172;449)	457 (275;540)	529 (498;557)	518 (328;560)	325 (168;419)	211 (149;301)	173 (126;295)	427 (365;476)	78	427 107 386

**[300-800]**												
**Neutrophils, count/µL [2500-12500]**	5430 (4351; 7665)	6403 (6023; 6970)	6336.5 (4571; 7976)	5733 (4671; 6813)	7232 (5623; 10528)	7682 (6150; 9322)	7392 (5045; 12208)	6755 (5592; 7655)	5264 (3052; 6867)	5468 (4533; 6941)	6200	5440 11840 6816
**Eosinophils, count/µL [0-1500]**	316 (160;441)	247 (182;491)	513 (369;915)	714 (481;846)	872 (540;989)	463 (333;789}	825 (652;1105)	734 (628;927)	847 (769;964)	505 (380;1068)	700	935 480 576
**Lymphocytes count/µL [1500-7000]**	3446 (2488; 4385)	2499 (1899; 3317)	4099 (3312; 4777)	3907 (3281; 5604)	3180 (2797; 3958)	3911 (3218; 4299)	3097 (2112; 4400)	2906 (2402; 3977)	2632 (2466; 3401)	2468 (1745; 3298)	2600	1870 3360 1920
**Monocytes, count/µL [0-850]**	434 (198;615)	257 (101;426)	226 (127;390)	163 (112;325)	564 (398;1117)	347 (291;504)	690 (464;759)	360 (236;602)	423 (272;423)	225 (149;296)	500	255 320 288

Data are median (interquartile range) except for data at Month 60, which are the values for individual cats. C, control group; HS, high salt group.

Hbg, hemoglobin; Htc, hematocrit.

Supplementary Table 2. Plasma biochemistry in aged cats fed a control or high-salt diet for up to 60 months

Variable [Reference interval]	Month 0		Month 12		Month 24		Month 36		Month 48		Month 60	
	C<br>N = 10	HS<br>N = 10	C<br>N = 10	HS<br>N = 10	C<br>N = 8	HS<br>N = 8	C<br>N = 7	HS<br>N = 8	C<br>N = 3	HS<br>N = 7	C<br>N = 1	HS<br>N = 3
Urea<br>mg/dL<br>[39.6-67.9]	50.8<br>(45.8;54.4)	52.6<br>(43.1;56.9)	41.1<br>(35.4;44.7)	44.1<br>(39.0;45.7)	48.4<br>(44.6;57.5)	45.1<br>(39.2;50.8)	57.7<br>(48.1;69.1)	49.6<br>(41.3;64.0)	45.7<br>(45.7;61.0)	46.9<br>(36.9;57.2)	61.3	34.2<br>39.0<br>74.5
Urea<br>mmol/L<br>[6.6-11.3]	8.5<br>(7.6;9.1)	8.8<br>(7.2;9.5)	6.9<br>(5.9 ;7.5)	7.4<br>(6.5;7.6)	8.1<br>(7.4;9.6)	7.5<br>(6.5;8.5)	9.6<br>(8,0;11.5)	8.3<br>(6.9;10.7)	7.6<br>(7.6;10.2)	7.8<br>(6.7;10.1)	10.2	5.7<br>6.5<br>12.4
Creatinine<br>mg/dL<br>[1.01-2.35]	1.40<br>(1.34;1.45)	1.50<br>(1.31;1.60)	1.58<br>(1.47;1.72)	1.60<br>(1.51;1.82)	1.31<br>(1.26;1.46)	1.45<br>(1.31;1.59)	1.25<br>(1.06;1.43)	1.29<br>(1.18;1.39)	1.24<br>(1.11;1.38)	1.56<br>(1.34;1.66)	1.43	1.19<br>1.57<br>1.91
Creatinine<br>µmol/L<br>[89.0-207.0]	123.8<br>(118.4;<br>128.4)	133.0<br>(115.5;<br>141.6)	139.4<br>(129.9;<br>151.7)	141.2<br>(133.3;<br>160.8)	116.2<br>(111.4;<br>129.1)	127.9<br>(116.1;<br>140.3)	110.8<br>(93.6;<br>126.6)	113.7<br>(104.7;<br>122.6)	109.6<br>(98.6;<br>122.3)	132.9<br>(110.7;<br>145.5)	126.4	105.4<br>138.5<br>168.8
Sodium<br>mEq/L<br>[153-161]	153<br>(153;154)	152<br>(150;155)	154<br>(153;155)	155<br>(154;156)	151<br>(151;153)	151.5<br>(151;152)	156<br>(154;157)	154<br>(153;156)	152<br>(151;154])	151<br>(150;153)	153	151<br>154<br>149
Potassium<br>mEq/L<br>[3.3-4.2]	4.3<br>(4.1;4.6)	4.3<br>(4.0;4.4)	3.9<br>(3.8;4.01)	3.9<br>(3.6;4.0)	4.2<br>(4.0;4.4)	4.2<br>(4.1;4.4)	4.1<br>(4.0;4.3)	4.5<br>(4.1;4.6)	4.0<br>(3.7;4.3)	4.4<br>(4.2;4.5)	4.1	4.1<br>4.2<br>4.0
Chloride<br>meq/L<br>[120-127]	119<br>(117;120)	120<br>(118;121)	116<br>(115;118)	117<br>(116;117)	118<br>(117;118)	117<br>(116;117)	121<br>(120;122)	118<br>(117;119)	121<br>(121;122)	119<br>(119;121)	124	117<br>122<br>125
Total CO_2<br>mEq/L<br>[15-21]	20<br>(20;21)	20<br>(19.25;20)	22<br>(20.25;22)	20<br>(20;21)	19<br>(18.75;19.<br>25)	19.5<br>(18.75;20)	20<br>(19.5;21)	20.5<br>(19.75;22)	15<br>(15;16.5)	17<br>(17;17.5)	17	18<br>17<br>12
Calcium<br>mg/dl<br>[9.6-11.6]	9.94<br>(9.76;<br>10.11)	9.80<br>(9.56;<br>9.94)	9.98<br>(9.86;<br>10.08)	9.82<br>(9.79;<br>10.20)	10.30<br>(10.05;<br>10.63)	10.36<br>(10.19;<br>10.66)	9.90<br>(9.58;<br>10.26)	10.04<br>(9.80;<br>10.46)	9.62<br>(9.52;9.86)	9.78<br>(9.56;<br>10.03)	9.22	10.62<br>9.74<br>9.50

Calcium, mmol/L [2.40-2.90]	2.48 (2.44;2.52)	2.45 (2.38;2.48)	2.49 (2.46;2.52)	2.45 (2.44;2.54)	2.57 (2.51;2.65)	2.58 (2.54;2.66)	2.47 (2.39;2.56)	2.5 (2.45;2.61)	2.4 (2.38;2.46)	2.43 (2.37;2.49)	2.3	2.65<br>2.43<br>2.37
Phosphate mg/dL [3.4-6.5]	3.98 (3.73;4.46)	3.77 (3.56;4.38)	4.19 (4.11;4.50)	4.63 (3.94;4.82)	4.19 (3.88;4.63)	4.31 (4.15;4.79)	4.12 (3.89;4.25)	4.62 (4.22;4.82)	4.34 (4.03;4.46)	4.76 (4.34;4.90)	4.53	5.02<br>4.46<br>4.71
Phosphate mol/L [1.10-2.10]	1.29 (1.2;1.44)	1.21 (1.15;1.41)	1.35 (1.32;1.45)	1.5 (1.27;1.55)	1.35 (1.25;1.49)	1.39 (1.34;1.55)	1.33 (1.25;1.37)	1.49 (1.36;1.55)	1.4 (1.3;1.44)	1.52 (1.33;1.57)	1.46	1.62<br>1.44<br>1.52
Albumin g/L [27.0-39.0]	34.20 (33.32; 35.27)	32.25 (31.18; 33.7)	30.45 (29.95; 30.98)	30.05 (28.70; 31.9)	32.55 (30.5; 33.05)	32.40 (31.85; 34.92)	30.10 (28.8; 33.55)	32.80 (30.55; 35.15)	30.10 (29.85; 31.7)	31.70 (31.0; 32.75)	25.00	33.1<br>28.3<br>27.8
Total protein g/dL [6.50-8.5]	7.55 (7.30;7.67)	7.13 (6.86;7.34)	6.99 (6.84;7.39)	6.88 (6.57;7.21)	7.74 (7.49;8.31)	7.54 (7.19;7.61)	8.08 (7.63;8.19)	7.59 (7.37;7.88)	8.00 (7.21;8.04)	7.59 (7.55;7.68)	7.43	7.79<br>7.25<br>73.40

Data are median (interquartile range) except for data at Month 60, which are the values for individual cats.

C, control group; CO_2, carbon dioxide; HS, high salt group.

Supplementary Table 3. Two-dimensional tissue Doppler imaging variables in aged cats fed a control or high-salt diet

Variable	Month 0		Month 12		Month 24		Month 36		Month 48		Month 60		Rate of change per month (time effect) (95% CI)		Difference between groups in rate of change (95% CI)
	C N = 10	HS N = 10	C N = 10	HS N = 10	C N = 8	HS N = 8	C N = 7	HS N = 8	C N = 3	HS N = 7	C N = 1	HS N = 3	C N = 10	HS N = 10	
**Radial motion of the LVFW**															
HR, beats/min	175 (164;188)	170 (163;180)	168 (159;178)	180 (178;184)	169 (162;177)	173 (164;192)	191 (173;193)	164 (148;192)	181 (170;188)	178 (151;202)	158	188 171 165	0.12 (−0.26; 0.50) **P=.52**	0.053 (−0.27; 0.38) **P=.75**	0.070 (−0.43; 0.57) **P=.78**
Systolic radial MVG, cm/s	2.2 (1.9;2.8)	2.1 (1.8;2.4)	2.3 (2.0;3.0)	2.7 (2.6;2.9)	2.5 (2.2;2.8)	2.8 (2.3;2.9)	2.3 (2.1;2.8)	2.3 (1.9;2.5)	1.9 (1.9;2.3)	2.2 (2.2;2.5)	2.4	2.3 3.1 2.2	0.0039 (−0.0083; 0.016) **P=.53**	0.0040 (−0.0063; 0.014) **P=.44**	−0.000087 (−0.016; 0.016) **P=.99**
E:A ratio below endocardium	1.51 (1.38;1.67)	1.56 (1.51;1.75)	1.74 (1.42;1.91)	1.39 (1.33;1.54)	1.88 (1.44;2.18)	1.24 (1.04;1.41)	1.44 (1.16;1.67)	1.24 (1.08;1.59)	1.48 (0.88;1.68)	1.1 (0.57;1.88)	1.41	1.24 0.51 1.19	−0.00066 (−0.0089;	−0.0094 (−0.017;	0.0087 (−0.0023;

													0.0076)<br>**P=.87**	−0.0022)<br>**P=.012**	0.020)<br>**P=.12**
E:A ratio below epicardium	1.54<br>(1.2;2.12)	1.61<br>(1.28;1.88)	2.03<br>(1.55;2.83)	1.47<br>(1.29;1.87)	2.29<br>(1.54;3.54)	1.36<br>(1.11;1.88)	2.37<br>(1.57;3.17)	1.4<br>(0.81;1.63)	1.89<br>(1.23;2.22)	0.8<br>(0.68;1.24)	1.42	1.63<br>0.57<br>1.32	0.018<br>(−0.0026;<br>0.039)<br>**P=.085**	−0.019<br>(−0.037;<br>−0.00090)<br>**P=.040**	0.037<br>(0.0096;<br>0.065)<br>**P=.009**
**Longitudinal motion of the LVFW**															
HR, beats/min	179<br>(166;190)	166<br>(163;1816)	169.5<br>(158;1796)	182<br>(169;193)	185<br>(161;197)	178<br>(167;186)	169<br>(167;179)	187.5<br>(179;193)	163<br>(162;1744)	180<br>(156;192)	185	174<br>174<br>173	−0.15<br>(−0.59;<br>0.28)<br>**P=.49**	0.29<br>(−0.079;<br>0.66)<br>**P=.12**	−0.44<br>(−1.01;<br>0.13)<br>**P=.13**
Systolic MVG base-apex, cm/s	2.1<br>(1.7;2.4)	2.15<br>(1.93;2.7)	2.2<br>(1.65;3.02)	2.15<br>(1.83;3.35)	2.4<br>(2.05;2.65)	2.45<br>(2.22;2.87)	2.5<br>(1.95;2.85)	2.8<br>(2.22;3.02)	2.1<br>(2.05;2.55)	2.6<br>(2.3;2.8)	2.2	1.2<br>2.8<br>4.1	0.012<br>(−0.0048;<br>0.030)<br>**P=.15**	−0.0022<br>(−0.017;<br>0.012)<br>**P=.76**	0.015<br>(−0.0079;<br>0.037)<br>**P=.20**
E:A ratio at the base	1.51<br>(1.19;1.72)	1.45<br>(1.24;1.84)	1.33<br>(1.1;1.76)	1.32<br>(1.23;1.72)	1.19<br>(0.96;1.59)	1.43<br>(0.98;1.56)	0.51<br>(0.46;0.94)	0.86<br>(0.43;1.45)	0.55<br>(0.52;1.18)	1.07<br>(0.64;2.35)	0.64	1.55<br>0.27<br>0.65	−0.021<br>(−0.035;<br>−0.0076)<br>**P=.0028**	−0.0064<br>(−0.018;<br>0.0048)<br>**P=.26**	−0.015<br>(−0.032;<br>0.0028)<br>**P=.098**

E:A ratio at the apex	1.73 (1.29;3.24)	2.19 (1.97;2.47)	1.71 (1.26;3.05)	2.94 (1.89;3.73)	2.99 (1.23;4.5)	2.18 (1.57;2.62)	1.03 (0.56;2)	0.99 (0.37;2.1)	0.76 (0.51;1.85)	2.22 (0.64;3.69)	0.77	3.83 0.19 1.11	−0.0088 (−0.088; 0.070) **P=.83**	−0.047 (−0.11; 0.019) **P=.16**	0.038 (−0.065; 0.14) **P=.46**
**Longitudinal motion of the IVS**															
HR, beats/min	182 (152;198)	176 (167;186)	187 (156;197)	186 (176;197)	181 (168;189)	188 (179;202)	184 (161;190)	173 (165;182)	187 (184;191)	176 (166;196)	185	201 176 178	0.19 (−0.20; 0.58) **P=.34**	0.11 (−0.22; 0.44) **P=.52**	0.081 (−0.43; 0.59) **P=.75**
S wave at the base, cm/s	4.6 (4.0;7.3)	5.7 (5.0;6.5)	5.9 (4.4;7.3)	6.7 (5.6;7.1)	5.8 (4.8;6.5)	6.8 (5.9;7.0)	5 (4.7;7.1)	5.8 (4.4;6.4)	4.8 (4.5;5.5)	5.5 (4.5;6.2)	3.8	5.9 5.1 3.9	0.020 (0.0028; 0.038) **P=.024**	−0.0095 (−0.024; 0.0052) **P=.20**	0.030 (0.0069; 0.052) **P=.012**
E:A ratio at the base	1.27 (1.17;1.32)	1.18 (0.79;1.27)	1.11 (1.03;1.26)	1.20 (0.77;1.36)	1.22 (0.91;1.35)	0.65 (0.49;1.30)	0.59 (0.52;1.26)	0.54 (0.4;1.45)	0.59 (0.5;0.83)	0.43 (0.41;0.94)	0.31	1.57 0.32 0.49	−0.0067 (−0.014; 0.00042) **P=.065**	−0.010 (−0.016; −0.0042) **P=.001**	0.0035 (−0.0058; 0.013) **P=.45**

Data are median (interquartile range) except for data at Month 60, which are the values for individual cats. The rate of change per month is the slope estimate from linear mixed models with time between Month 0 and Month 48 as a continuous variable. The difference between groups is the slope estimate contrast (control minus high-salt group).

C, control-diet group; CI, confidence interval; E, peak velocity of early diastolic transmitral flow; A, peak velocity of late diastolic transmitral flow; HR, heart rate; HS, high-salt diet group; IVS, interventricular septum; LVFW, left ventricular free wall; MVG, myocardial velocity gradient.

Supplementary Table 4. Renal resistive indexes in aged cats fed a control or high-salt diet for up to 60 months

Variable	Month 0		Month 12		Month 24		Month 36		Month 48		Month 60		Rate of change per month (time effect) (95% CI)		Difference between groups in rate of change (95% CI)
	**C N = 10**	**HS N = 10**	**C N = 10**	**HS N = 10**	**C N = 8**	**HS N = 8**	**C N = 7**	**HS N = 8**	**C N = 3**	**HS N = 7**	**C N = 1**	**HS N = 3**	**C N = 10**	**HS N = 10**	
LRA	0.69 (0.68; 0.69)	0.68 (0.67; 0.70)	0.67 (0.64; 0.72)	0.71 (0.66; 0.74)	0.64 (0.62; 0.66)	0.64 (0.61; 0.67)	0.66 (0.64; 0.66)	0.64 (0.63; 0.66)	0.66 (0.65; 0.67)	0.67 (0.64; 0.69)	0.69	0.63<br>0.69<br>0.69	−0.00103 (−0.00191; −0.000146) **P = .023**	0.00085 (−0.00160; −0.000102) **P = .027**	−0.000175 (−0.00133; 0.00098) **P = .76**
LIA	0.69 (0.68; 0.69)	0.68 (0.65; 0.69)	0.66 (0.64; 0.7)	0.64 (0.63; 0.65)	0.66 (0.63; 0.66)	0.64 (0.62; 0.67)	0.66 (0.64; 0.68)	0.66 (0.64; 0.67)	0.63 (0.63; 0.66)	0.65 (0.64; 0.66)	0.69	0.65<br>0.66<br>0.64	−0.000749 (−0.00148; −0.0000209) **P = .044**	−0.000175 (−0.00080; 0.000449) **P = .58**	−0.000574 (−0.00153; 0.000385) **P = .24**
RRA	0.69 (0.66; 0.69)	0.68 (0.65; 0.69)	0.71 (0.66; 0.73)	0.69 (0.62; 0.71)	0.65 (0.63; 0.67)	0.64 (0.62; 0.65)	0.65 (0.64; 0.66)	0.64 (0.63; 0.65)	0.67 (0.65; 0.68)	0.68 (0.67; 0.68)	0.70	0.66<br>0.72<br>0.65	−0.000835 (−0.00165; −0.0000189) **P = .045**	−0.000373 (−0.00107; 0.000324) **P = .29**	−0.000462 (−0.00153; 0.000611) **P = .39**

RIA	0.68<br>(0.66;<br>0.69)	0.67<br>(0.66;<br>0.69)	0.67<br>(0.63;<br>0.7)	0.66<br>(0.62;<br>0.67)	0.65<br>(0.63;<br>0.66)	0.64<br>(0.61;<br>0.67)	0.66<br>(0.65;<br>0.69)	0.64<br>(0.6;<br>0.65)	0.67<br>(0.64;<br>0.68)	0.67<br>(0.64;<br>0.68)	0.70	0.64<br>0.67<br>0.65	−0.000319<br>(−0.00113;<br>0.000491)<br>**P = .43**	−0.000764<br>(−0.00145;<br>−0.0000751)<br>**P = .030**	0.000444<br>(−0.000619;<br>0.00151)<br>**P = .41**

Data are median (interquartile range) except for data at Month 60, which are the values for individual cats. The slope estimate is the rate of change per month from linear mixed models with time between Month 0 and Month 48 as a continuous variable. The slope estimate contrast is the comparison between diets in rates of change (control minus high-salt group). The resistive index of the renal artery and interlobar arteries was calculated from the formula: (peak systolic velocity - end-diastolic velocity) / peak systolic velocity.

C, control group; CI, confidence interval; HS, high-salt group; LRA, left renal artery; LIA, left interlobar artery; RI, resistive index; RRA, right renal artery; RIA, right interlobar artery.

Supplementary Table 5. Case review of serial biochemistry and spot USG data, and key postmortem findings

Group / cat ID and sex	Min-max range during study and trend for changes over time				GFR at baseline and every 12 mths, mL/min/kg	Time in study, mths	Reason for leaving study	Evidence of CKD during study based on biochemistry and BW	Postmortem macroscopic and microscopic findings
	Serum creatinine, µmol/L<br><br>Trend	Spot USG<br><br>Trend	Serum urea, mmol/L<br><br>Trend	BW, kg<br><br>Trend					
C / 4<br>Male neutered	128-169<br><br>No	1.045-1.060<br><br>No	6.7-9.1<br><br>No	4.6-4.8<br><br>No	1.9; 1.8; 1.5	25.3	Euthanized owing to a diagnosis of lymphoma	No biochemical evidence of CKD	Epitheliotropic small cell lymphoma of the esophagus<br><br>Mild multifocal chronic lymphoplasmacytic tubulointerstitial nephritis
C / 5<br>Female neutered	83-133<br><br>No	1.027-1.051<br><br>No	7.0-8.4<br><br>No	4.3-3.7<br><br>↓	2.5; 1.6; 2.1; 2.3; 2.7	59.7	Euthanized owing to a diagnosis of lymphoma	No biochemical evidence of CKD	Moderate to marked diffuse chronic lymphoplasmacytic enteritis<br><br>Chronic diffuse moderate to marked lymphoplasmacytic colitis<br><br>No evidence of renal pathology
C / 7<br>Male neutered	116.2-164.0<br><br>No (3 of 6 values >140)	1.015-1.050<br><br>↓ from 24 months	7.5-13.7<br><br>↑ after 12 months	5.7-4.0<br><br>↓ after 12 months	1.8; 2.0; 1.3; 1.1	38.2	Died	Deteriorating renal function after 12 months with reduced USG, rising plasma creatinine and urea concentration and loss of BW<br><br>Declining GFR after 12 months supports that conclusion	Death attributed to a diffuse lymphoma of the intestinal wall (small intestine and colon)<br><br>Moderate to marked chronic bilateral interstitial nephritis leading to parathyroid gland hyperplasia
C / 8<br>Male neutered	99.6-128.9<br><br>No	1.029-1.040<br><br>No	5.7-8.7<br><br>No	4.4-4.9<br><br>No	1.7; 1.7; 1.6; 1.9; 1.7	51.0	Excluded for hypertension	No biochemical evidence of CKD	No postmortem examination
C / 16<br>Male neutered	135.6-156.5<br><br>↑ over 6 mths then stable at >140	1.040-1.058<br><br>No	7.0-8.5<br><br>↓	5.7-6.5<br><br>No	1.2; 2.3	17.6	Excluded for diabetes mellitus	No clinical evidence of deteriorating kidney function over 12 months	No postmortem examination

Group / cat ID and sex	Min-max range during study and trend for changes over time				GFR at baseline and every 12 mths, mL/min/kg	Time in study, mths	Reason for leaving study	Evidence of CKD during study based on biochemistry and BW	Postmortem macroscopic and microscopic findings
	**Serum creatinine, µmol/L**<br><br>**Trend**	**Spot USG**<br><br>**Trend**	**Serum urea, mmol/L**<br><br>**Trend**	**BW, kg**<br><br>**Trend**					
C / 19 Male neutered	115.0-153.0<br><br>↑ over first 12 mths then declined	1.016-1.025<br><br>No Persistently low	8.7-12.7<br><br>No	3.4-4.2<br><br>↓ in last 2 years; stable prior to that	2.2; 2.4; 1.6; 1.2; 1.1	60.0	Completed study<br><br>Euthanized owing to a diagnosis of CKD 1.9 months after study end	Low USG and rising creatinine over first 12 mths might suggest early CKD but this did not progress<br><br>Falling GFR from 24 mths supports this	Marked multifocal lymphoplasmacytic chronic interstitial nephritis with nephrocalcinosis<br><br>Moderate to marked diffuse chronic lymphoplasmacytic enteritis<br><br>Moderate diffuse lymphoplasmacytic gastritis
C / 21 Female neutered	110.8-132.9<br><br>No	1.025-1.049<br><br>↓ from 6 to 36 mths	8.5-11.0<br><br>No	4.3-5.7<br><br>↓ between 24 and 36 mths	1.9; 2.5; 1.7; 1.7	39.5	Euthanized owing to rapid weight loss and acute kidney injury subsequent to a dental procedure	No strong evidence of CKD (two USG values <1.030)	No postmortem examination
C / 22 Female neutered	87.5-128.7<br><br>No	1.015-1.030<br><br>↓ from 12 to 36 mths	5.2-6.6<br><br>No	5.3-6.0<br><br>No	2.1; 1.8; 1.9; 2.1	39.1	Euthanized owing to chronic enteritis	Decreasing USG but no other indication of early CKD	Moderate to marked diffuse chronic lymphoplasmacytic enteritis<br><br>Moderate diffuse lymphoplasmacytic gastritis<br><br>Marked hydropic degeneration of centrilobular hepatocytes associated with marked intrahepatocytic and intracanalicular cholestasis<br><br>Minimal to mild multifocal lymphoplasmacytic chronic interstitial nephritis<br><br>Pancreatic amyloidosis of the islets of Langerhans

Group / cat ID and sex	Min-max range during study and trend for changes over time				GFR at baseline and every 12 mths, mL/min/kg	Time in study, mths	Reason for leaving study	Evidence of CKD during study based on biochemistry and BW	Postmortem macroscopic and microscopic findings
	**Serum creatinine, µmol/L**<br>**Trend**	**Spot USG**<br>**Trend**	**Serum urea, mmol/L**<br>**Trend**	**BW, kg**<br>**Trend**					
C / 23 Female neutered	113.0-145.7<br>No	1.016-1.058<br>No	6.5-9.6<br>No	3.0-4.3<br>↓	2.1; 2.3; 2.2; 2.0	37.9	Euthanized owing to lymphoma	No evidence of deteriorating renal function. USG were >1.040 except for a single reading of 1.016 at Month 36	Moderate diffuse lymphoplasmacytic gastritis<br>Chronic diffuse lymphoplasmacytic and neutrophilic enteritis, severe, chronic fibrosis<br>Chronic diffuse moderate to marked lymphoplasmacytic colitis<br>Marked fibrosis of the media and adventitia of the centrilobular splenic arteries<br>Moderate to marked diffuse chronic interstitial nephritis
C / 24 Female neutered	114.9-121.7<br>No	1.042-1.049<br>No	5.1-8.5<br>No	5.3-5.8<br>No	1.8; 1.9	13.6	Euthanized owing to fibrosarcoma	No evidence of deteriorating renal function	Subcutaneous interscapular fibrosarcoma<br>Moderate to marked thickening of glomeruli basement membranes and glomerulosclerosis<br>Mild renal interstitial fibrosis and lymphocytic infiltration
HS / 6 Female neutered	97.6-128.8<br>No	1.037-1.064<br>No	5.0-7.2<br>No	5.4-4.7<br>↓ but not progressive	2.0; 1.7; 1.8; 2.1; 1.8	52.3	Excluded owing to diabetes mellitus	No biochemical evidence of CKD	No postmortem examination
HS / 9 Female neutered	53.1-154.5<br>↓ after 6 mths	1.015-1.047<br>↓ after 12 mths	6.5-10.8<br>↑ after 12 mths	2.6-4.1<br>↓ after 12 mths	1.7; 1.3; 1.6; 2.6; 2.6	49.5	Excluded owing to hyperthyroidism	Changes in blood and urine variables and GFR explained by hyperthyroidism; no evidence of co-existing kidney disease	No postmortem examination
HS / 10 Female neutered	90.3-142.5<br>↓ after 12 mths	1.026-1.041<br>No (2 values <1.030)	7.0-8.5<br>No	3.2-3.8<br>No	2.3; 2.1; 1.8; 2.2	44.4	Euthanized owing to nasal tumor	No biochemical evidence of CKD	Infiltrating and osteolytic mucosecretory nasal adenocarcinoma<br>Mild bilateral lymphoplasmacytic tubulointerstitial nephritis

Group / cat ID and sex	Min-max range during study and trend for changes over time				GFR at baseline and every 12 mths, mL/min/kg	Time in study, mths	Reason for leaving study	Evidence of CKD during study based on biochemistry and BW	Postmortem macroscopic and microscopic findings
	**Serum creatinine, µmol/L**<br><br>**Trend**	**Spot USG**<br><br>**Trend**	**Serum urea, mmol/L**<br><br>**Trend**	**BW, kg**<br><br>**Trend**					
HS / 11 Male neutered	137.1-156.2<br><br>No (3 of 4 values >140)	1.027-1.038<br><br>No	7.6-10.4<br><br>↓	5.1-5.5<br><br>No	1.9; 2.1	13.4	Died	No clinical evidence of deteriorating kidney function	Mild multifocal lymphoplasmacytic chronic interstitial nephritis
HS / 12 Male neutered	139.6-250.2<br><br>No (borderline azotemia from time 0; overt azotemia at 48 mths)	1.033-1.016<br><br>↓	7.8-13.4<br><br>↑ from 12 mths	4.5-4.0<br><br>↓ over last 2 years	1.7; 1.2; 1.2; 1.2; 1.2	50.5	Excluded owing to development of CKD<br><br>Adopted	CKD evidenced by azotemia (plasma creatinine mostly borderline but increased to 250 µmol/L at Month 48) with low USG<br><br>Declining GFR from 6 months is in line with declining renal function	No postmortem examination
HS / 13 Female neutered	99.4-127.6<br><br>No	1.022-1.058<br><br>No	4.4-7.0<br><br>No	3.4-4.5<br><br>No ↑ from 24 mths	1.8; 1.9; 2.0; 1.6; 1.7; 1.3	60.0	Completed study<br><br>3.5 years after study completion was euthanized owing to a diagnosis of CKD complicated with pneumonia	No biochemical evidence of CKD - USG remained ≥1.035 between 12 and 60 months and creatinine never >140 even when GFR was measured at 1.3 mL/min/kg which was the last measurement	Moderate to marked chronic interstitial nephritis associated with muscle wasting and anemia, and multifocal nephrocalcinosis<br><br>Mild to moderate parathyroid gland hyperplasia<br><br>Moderate multifocal medial mucinosis in aorta, focal marked fibrosis of the myocardium, and marked hypertrophy of the media of the pulmonary arterioles<br><br>Subacute suppurative pneumonia
HS / 14 Female neutered	119.6-162.3<br><br>No (3 of 8 values >140)	1.027-1.060<br><br>No	6.3-7.8<br><br>No	3.3-3.7<br><br>No	2.5; 1.9; 1.8; 2.0; 2.2; 1.5	60.0	Completed study<br><br>Euthanized 1.6 years after study end owing to pulmonary carcinoma	No biochemical evidence of CKD by the end of the study	Bronchioloalveolar lung carcinoma with pleural carcinomatosis<br><br>Moderate chronic interstitial fibrosis and inflammation with multifocal mild glomerulosclerosis

Group / cat ID and sex	Min-max range during study and trend for changes over time				GFR at baseline and every 12 mths, mL/min/kg	Time in study, mths	Reason for leaving study	Evidence of CKD during study based on biochemistry and BW	Postmortem macroscopic and microscopic findings
	Serum creatinine, μmol/L<br><br>Trend	Spot USG<br><br>Trend	Serum urea, mmol/L<br><br>Trend	BW, kg<br><br>Trend					
HS / 17 Male neutered	131-177.1<br><br>No (6 of 7 values including baseline >140)	1.017-1.036<br><br>Declined from1.030 in last 2 years	7.6-10.2<br><br>No	4.6-5.6<br><br>No	1.6; 1.0; 1.1; 1.2; 1.2	55.9	Excluded owing to megacolon<br><br>Euthanized 4.4 mths after exclusion owing to megacolon	USG persistently below 1.035 and declined between 24 to 48 months and creatinine values borderline for azotemia possibly indicative of early-stage CKD<br><br>This is supported by low GFR measurements from 12 months	Megacolon<br><br>Focal renal lesions and marked multifocal glomerulosclerosis and corticomedullary interstitial lymphocytic/plasmacytic inflammation, accompanied by bilateral parathyroid hyperplasia suggestive of CKD
HS / 18 Male neutered	109.6-132.5<br><br>No	1.041-1.058<br><br>No	6.6-9.4<br><br>No	5.0-5.8<br><br>↑ over first 6 mths	1.8; 1.7	21.2	Died	No biochemical evidence of CKD	Death due to meningioma<br><br>Marked diffuse cardiac lipomatosis<br><br>Moderate thickening of glomeruli basement membranes<br><br>Mild renal interstitial fibrosis and lymphocytic infiltration<br><br>Moderate thickening of medial walls of accurate arteries
HS / 25 Male neutered	112.0-168.8<br><br>↑ after 36 mths	1.013-1.044<br><br>↓ over whole 60 mths	7.1-12.4<br><br>↑ in alignment with creatinine	4.1-4.8<br><br>↓ after 24 mths	2.1; 1.6; 1.4; 1.6; 1.0; 0.9	60.0	Completed study<br><br>Euthanized 3.6 mths after study end	Increasing plasma creatinine concentrations and decreasing USG accompanied by decrease in BW over the last 2-3 years, together suggestive of early-stage CKD<br><br>This is supported by decline in GFR particularly at after 36 months	Marked renal interstitial inflammation and fibrosis, glomerulosclerosis with thickening of Bowman's capsule and multiple areas of nephrocalcinosis in medulla<br><br>Parathyroid gland hyperplasia

BW, body weight; C, control; CKD, chronic kidney disease; GFR, glomerular filtration rate; HS, high-salt; ID, identification; mth, month; USG, urine specific gravity.

Supplementary Table 6. Effect sizes for pairwise comparisons between diets at each timepoint

Variable	Interaction Diet x Time<br>P value	Effect size for differences between diet groups (C minus HS diet) at each time point (P-value)				
		Month 0	Month 12	Month 24	Month 36	Month 48
**Indicators of renal function**						
GFR, bodyweight adjusted	0.3051	0.02<br>(.97)	1.22<br>(.03)	0.48<br>(.42)	−0.05<br>(.93)	0.54<br>(.49)
Plasma creatinine*	0.0022	0.02<br>(.20)	0.57<br>(.50)	0.21<br>(.09)	−0.10<br>(.67)	0.17<br>(.20)
**Blood pressure**						
Systolic arterial BP	0.6374	−0.3<br>(.55)	−0.5<br>(.34)	0.2<br>(.73)	0.4<br>(.55)	0.4<br>(.64)
**Echocardiography: M mode variables**						
LVDd, mm	0.6991	0.50<br>(.41)	0.61<br>(.31)	−0.17<br>(.80)	0.57<br>(.39)	−0.21<br>(.80)
LVDs mm	0.4219	−0.09<br>(.88)	0.81<br>(.19)	−0.40<br>(.55)	−0.12<br>(.86)	0.30<br>(.72)
LVFWd, mm	0.0766	1.2<br>(.09)	−0.1<br>(.86)	0.9<br>(.21)	1.8<br>(.02)	1.1<br>(.20)

LVFWs, mm	0.2073	0.6<br>(.33)	0.3<br>(.59)	1.2<br>(.08)	1.7<br>(.02)	2.0<br>(.03)
IVSd, mm	0.4033	−0.22<br>(.75)	−0.11<br>(.87)	0.87<br>(.25)	0.76<br>(.31)	0.04<br>(.96)
IVSs	0.1699	−0.23<br>(.76)	−0.23<br>(.76)	0.96<br>(.22)	1.1<br>(.16)	0.07<br>(.94)
FS, %	0.2184	0.44<br>(.53)	−0.72<br>(.30)	0.53<br>(.48)	0.40<br>(.59)	−0.57<br>(.52)
**Echocardiography: two-dimensional variables**						
LA:Ao	0.764	0.16<br>(.75)	0.52<br>(.28)	0.39<br>(.46)	−0.05<br>(.93)	−0.49<br>(.51)
Subaortic IVSd, mm	0.6996	−0.43<br>(.58)	0.09<br>(.90)	0.52<br>(.52)	0.13<br>(.87)	−0.30<br>(.75)
**Doppler variables**						
Peak aortic flow velocity, m/s†	0.6291	−0.25<br>(.71)	−0.11<br>(.86)	−1.01<br>(.15)	−0.14<br>(.84)	−0.87<br>(.31)
Mitral E:A ratio	0.2421	0.27<br>(.77)	−0.35<br>(.70)	−1.10<br>(.25)	0.29<br>(.75)	−0.72<br>(.49)
IVRT, ms	0.723	0.04<br>(.93)	−0.53<br>(.25)	−0.29<br>(.58)	0.33<br>(.53)	0.21<br>(.77)

**TDI: radial motion of the LVFW**						
HR, beats/min†	0.0977	0.28 (.59)	−0.88 (.10)	−0.59 (.31)	0.80 (.18)	−0.48 (.54)
Systolic radial MVG, cm/s	0.7098	0.58 (.28)	−0.15 (.78)	−0.03 (.96)	0.57 (.35)	−0.07 (.93)
E:A ratio below endocardium	0.0154	−0.89 (.20)	1.00 (.15)	1.15 (.12)	0.22 (.76)	0.51 (.57)
E:A ratio below epicardium†	0.0811	−0.57 (.35)	0.61 (.32)	0.80 (.23)	1.00 (.14)	1.43 (.10)
**TDI: longitudinal motion of the LVFW**						
HR, beats/min	0.275	0.61 (.23)	−0.55 (.28)	−0.19 (.73)	−0.56 (.34)	−0.86 (.26)
Systolic MVG base-apex, cm/s†	0.8125	−0.47 (.38)	−0.21 (.70)	−0.20 (.74)	0.37 (.55)	0.10 (.90)
E:A ratio at the base*	0.6215	0.02 (.45)	−0.07 (.54)	0.03 (.27)	−0.14 (.09)	−0.15 (.10)
E:A ratio at the apex*	0.8393	−0.18 (.27)	−0.20 (.22)	0.12 (.82)	0.07 (.56)	−0.25 (.27)
**TDI longitudinal motion of the IVS**						

HR, beats/min	0.6043	0.01<br>(.99)	−0.32<br>(.60)	−0.62<br>(.35)	0.52<br>(.44)	−0.21<br>(.81)
S wave at the base, cm/s	0.0155	1.30<br>(.18)	1.06<br>(.27)	1.41<br>(.16)	3.05<br>(.00)	3.10<br>(.01)
E:A ratio at the base	0.6713	0.13<br>(.85)	0.33<br>(.64)	1.02<br>(.17)	0.40<br>(.60)	1.08<br>(.24)

*Data were rank transformed and effect sizes were calculated according to r Rosenthal's methodology. †Data were log transformed and effect size was calculated by cohen methodology. Other data were not transformed, and effect size was calculated by cohen methodology.

d cohen effect sizes of 0.3, 0.5 and 0.8 are considered to be small, medium and large values, respectively. r Rosenthal's effect sizes of 0.1, 0.3 and 0.5 are considered to be small, medium and large values, respectively.

C, control-diet group; BP, blood pressure; HS, high-salt diet group; IVSd, interventricular thickness at end-diastole; LVFWd, left ventricular free wall thickness at end-diastole.
